# Metaheuristic aided structural topology optimization method for heat sink design with low electromagnetic interference

**DOI:** 10.1038/s41598-024-54083-z

**Published:** 2024-02-10

**Authors:** Musaddiq Al Ali, Masatoshi Shimoda, Brahim Benaissa, Masakazu Kobayashi, Tsunehiro Takeuchi, Ameer Al-Shawk, Sina Ranjbar

**Affiliations:** 1 Department of Advanced Science and Technology, Toyota Technological Institute, 2-12-1 Hisakata, Tenpaku-Ku, Nagoya, Aichi 468-8511 Japan; 2 Stellantis North America Headquarters, Auburn Hills, MI 48326 USA; 3 IMEC, Kaprldreef 75, 3001 Heverlee, Belgium

**Keywords:** MASTO method, Non-parametric optimization, Heat sink design, Electromagnetic interference, Multiphysics problem, Engineering, Mathematics and computing, Physics

## Abstract

This study investigates the application of the Metaheuristic Aided Structural Topology Optimization (MASTO) method as a novel approach to address the multiphysics design challenge of creating a heat sink with both high heat conductivity and minimal Electromagnetic Interference (EMI). A distinctive 2D layout with elongated fins is examined for electromagnetic traits, highlighting resonance-related EMI concerns. MASTO proves to be a valuable tool for navigating the complex design space, yielding thoughtfully optimized solutions that harmonize efficient heat dissipation with effective EMI control. By merging simulation findings with practical observations, this study underscores the potential of the MASTO method in achieving effective designs for intricate multiphysics optimization problems. Specifically, the method's capacity to address the complex interplay of heat transfer with convection and the suppression of electromagnetic emissions is showcased. Moreover, the study demonstrates the feasibility of translating these solutions into tangible outcomes through manufacturing processes.

## Introduction

The rising integration of high-speed electronics in applications such as smart vehicles and trains accentuates the need to enhance thermal management. Achieving goals of reduced weight and increased reliability requires continuous research into integrated systems, advanced heat dissipation techniques, and improved thermal conductivity. In this context, heat sinks remain a dependable, energy-efficient, and cost-effective solution for effective thermal management. Although metallic materials are often preferred for heat sinks due to their excellent conductivity and stability, their metal composition poses the potential challenge of electromagnetic interference (EMI)^1^. Moreover, the increasing of heat dissipation performance by elongating the fin structures of the heat sink makes the fins works as matched antenna with the resonant spurious electromagnetic waves.

EMI remain an undesirable characteristic in electronic and electrical engineering due to its harmful effect on the accuracy of data transmission^2^, and the electromagnetic absorption dosage for humans^3^. To address this problem, various approaches have been suggested, such as absorbing material^4^, electric grounding^5^, and shielding^6^ are utilized. However, in this research we are investigating the methodology of addressing the EMI elimination through the design phase. By addressing the heat sink design, the circulation of current in a heatsink that is caused by parasitic couplings between leaked power form electronic devices and the heatsink can be decreased significantly.

Topology optimization is one of the most powerful and effective methodologies for designing heat sinks^7–9^. This design approach proves particularly useful for solving the problem of weight reduction and ensuring reliability in high-speed and high-power electronic devices^10^. By incorporating simulation discretization as design variables in the multiphysics optimization problem, it becomes possible to simultaneously address both EMI and heat conductivity problems in heat sink design. This approach opens the door to discovering novel, cost-effective solutions that excel in dissipating heat efficiently while maintaining a lightweight profile and minimizing EMI.

This design approach originated from J. Maxwell's studies in 1869^11^, with later development by Michell establishing the foundation. Methods like Discretized Continuous Optimality Criterion (DCOC)^12^ and Solid Isotropic Material with Penalization (SIMP) gained traction. Techniques like Evolutionary Structural Optimization, Phase-field, and Level set were introduced. SIMP excelled in high thermal conductivity structure design^13,14^. The contemporary approach to optimizing high-heat-dissipation structures follows Bejan's volume-to-point method^15^. This method enhances overall thermal conductivity by considering shape, layout, size, and heat flow. Many adopt heat compliance minimization as the objective function. Qing et al. applied evolutionary structural optimization (ESO) to temperature-controlled points^16^, and Gersborg-Hansen et al. explored finite volume discretization^17^. Regarding heat sink design and topology optimization, mathematical algorithms are employed to enhance the thermal performance. This involves modifying the fin's shape and components to decrease heat flow resistance and increase the surface area. The result is a compact design, capable of managing high thermal loads and adaptable across different application areas, such as antenna^18^ and microelectronic mechanical system (MEMS) design^19^. Topology optimization gave the freedom to designers to combine multiphysics problems and attain innovative solutions^13,20–26^.

Our Multiphysics problem consists of two distinct parts. The first part focuses on minimizing the potential EMI of the heat sink during the design phase and addressing the challenges associated with achieving this objective. The challenges may be listed as the following.

The first challenge is defining EMI criteria. To minimize EMI, understanding "good antennas" is vital. Irrelevant aspects like materials, cost, and structure are excluded. Antenna quality is linked to impedance matching with the leak source, influenced by metallic shape variations. Impedance relates to an antenna's Radio Frequency (RF) resistance; shapes affect electrical traits. Leaked signals and potential harmonics are known from device specs, making impedance matching a viable objective criterion.

The second challenge is analyzing electromagnetic radiation numerically, demanding suitable methods like finite-difference time-domain, boundary element, and finite-element techniques to assess EMI in complex topological geometries. These methods provide detailed insights into electromagnetic properties, aiding EMI issue identification. Initially, finite difference was used for assessing irregular waveguides^27^. The finite difference in traditional form may lack the adaptation to simulate complex shapes, but some techniques was used to overcome this limitation such as the conformal mapping^28^. Soon after, the conformal mapping technique gain dominance for evaluation electromagnetic wave propagation for metallic structure^29^. The finite difference method evolved into FDTD, widely used in electromagnetic simulation. Conversely, finite-element method is versatile for various engineering and physics challenges, particularly multiphysics systems involving interactions like mechanical, thermal, and electrical behaviors. P. P. Silvester pioneered finite element modeling for complex electromagnetic structures, including arbitrary waveguides^30^. Here we chose the finite element method to evaluate the heat sink design. As such, it is imperative to use a dedicated suitable finite element system to ensure attaining sufficient evaluation of the electromagnetic radiation characteristics of the design antenna.

The third challenge involves the multiphysics objective function, which seeks a light, heat-conductive, EMI-minimal structure. Simplifying features lowers computation. The heat sink's EMI is measured by VSWR, the ratio of forward and backward waves on a line. Lower VSWR means more leakage. Higher VSWR is better for EMI-less heat sinks. VSWR evaluates telecom devices like phones, lines (WiFi, GPS, etc.), and soldering in RF switches. VSWR is measured by VNAs and meters. Theoretical VSWR ranges from 1 to ∞ for absolute mismatch (short/open circuit), lower VSWR indicating better impedance matching and more power reception. In RF industry, a good antenna's VSWR is typically < 1.3 to 2, while mismatched antennas exceed 10 (which is the particle implementation of industry). VSWR calculations consider transmission lines or waveguides. The FEM approach breaks these into small segments, solving Maxwell's equations for each, allowing electric and magnetic field determination along the line.

Returning to the heat dissipation maximization physics, we are adopting the Bejan approach^15^, with considering the minimization of the heat compliance. In this research, we are trying to attain, both, the maximization of the VSWR and the minimization of the main heat compliance for the heat sing during the design phase. As such the main objective function that satisfies the goal of designing heat sink with low EMI is chosen as the proportional function minimization of the heat compliance and the VSWR. The design is based on the multi-search approach that utilizes YUKI Metaheuristic search algorithm as the optimizer^31^.

With the clarification of the main optimization factors in the previous sections, we need to answer the key question which is how to perform the designing process for the multiphysics problem of heat and electromagnetic propagation. There are two major obstacles that impede the progress of our multiphysics optimization. The first obstacle lies in the difficulty of obtaining accurate derivatives through sensitivity analysis for complex multiphysics problems. The second major obstacle is the dependence of the final optimized design on the initial values, which significantly affects the ability to attain an optimal solution using traditional topology optimization methods^32,33^.

Initial values affect the final design in topology optimization. They can be material, boundary, or field. Initial values impact design and performance. Bad ones give poor solutions and good ones speed up accuracy. Strategies reduce initial value dependence. Adaptive algorithms change values during optimization, reaching optimal solutions. Other methods use fixed-level search like ESO and level set^34^.

In this study, we propose utilizing meta-heuristics to determine optimal initial values for the topology optimization process. Our Metaheuristic Structural Topology Optimization method (MASTO) employs two-level optimization using the YUKI metaheuristic optimization and the topology optimization with the Optimality Criteria (OC) search optimizer. Initially, we define initial design domains within the YUKI search space for topology optimization. We then evaluate heat dissipation performance, crucial for minimizing stored heat energy and maximizing Voltage Standing Wave Ratio (VSWR), through the YUKI algorithm. This process involves updating initial sets for subsequent design domains based on prior evaluations. Ultimately, we converge towards optimal initial design domain sets aligned with our primary objective. Our approach diverges from traditional single-point or single-level optimization by leveraging the initial dependency inherent in non-parametric optimization, turning it into a key asset for searching optimal solutions for complex multiphysics problems.

In order to clarify the method, and presenting the results in the best way possible, the rest of the paper is organized as the following: “Mathematical modelling of MASTO method for heat conduction structure under natural convection with low EMI” delineates the formulation of heat dissipation modelling, VSWR analysis, and introduces the MASTO optimization algorithm. Moving to “Numerical investigation”, we delve into numerical explorations concerning the design of a low EMI heat sink fin. This segment encapsulates both numerical and experimental validations. Ultimately, “Conclusion” encapsulates the conclusion.

## Mathematical modelling of MASTO method for heat conduction structure under natural convection with low EMI

Topology optimization falls within the realm of non-parametric optimization methodologies, with its primary objective being the realization of designs by tackling a numerically discretized representation of the design challenge.

This study focuses on employing topology optimization to simultaneously enhance heat transfer efficiency and reduce the generated EMI by minimizing heat compliance and maximizing the VSWR within a structural layout. Typically, topology optimization involves discretizing design variables (${\mathbf{x}}$) using finite element methods. In our research, we utilized linear triangular elements for electromagnetic simulations and bilinear rectangular structured mesh for heat transfer and convection simulations. A value of 1 signifies a solid element, whereas 0 represents a void element, as depicted in Eq. (1).

1 $${\mathbf{x}} = \left\{ {\begin{array}{ll}    1 & \quad {Design \; materials}  \\    0 & \quad {Void}  \\   \end{array} } \right.$$

In this section, we delve into the mathematical modeling of maximizing heat transfer while addressing heat compliance. Following that, we'll explore the mathematical modeling of electromagnetic propagation phenomena. Finally, we will discuss the optimization algorithm employed in our study.

### Mathematical modelling for maximizing heat conductivity for solid structures

For thermal dissipater optimization, the problem is reciprocal. Not optimizing original design domain, with ROI as heat source and boundaries as heat flow, the approach minimizes heat compliance. It uses distributed heat source and adiabatic boundaries, except ROI at zero. Enhancing conductivity minimizes thermal energy by reducing compliance. The analysis considers heat conduction and transfer from ROI to boundaries with convection. Only steady-state is examined. The equation is:

2 $$ {\mathbf{Q}} = {\mathbf{KT}} $$

where, ${\mathbf{Q}}$ is the nodal heat load, ${\mathbf{T}}$ nodal temperature, and is the ${\mathbf{K}}$ is heat conductivity matrix. Steady-state heat conduction topology optimization aims to identify an optimal structural layout that minimizes heat generation within a specified solid material volume. This optimization involves material distribution that seeks to lower the structure's temperature, thus enhancing thermal dissipation. As such, minimizing the heat potential capacity (i.e., heat compliance $C$) has chosen as the objective function (Eq. 3), with design variables determined by element state variables, i.e., density variables that indicate the presence (1) or absence (0) of materials.

3 $$ C = {\mathbf{Q}}^{T} {\mathbf{T}} $$

Moreover, in this research, we are considering the heat convection. The heat convection governing equation is the relation between the heat convection load ${\mathbf{Q}}_{Conv}$ and the relative difference of the ambient temperature above the surface of the solid design domain ${\mathbf{T}}_{\infty }$ and the solid design domain ${\mathbf{T}}_{s}$.

4 $$ {\mathbf{Q}}_{Conv} = A{\mathbf{k}}_{Conv} ({\mathbf{T}}_{s} - {\mathbf{T}}_{\infty } ) $$

where ${\mathbf{k}}_{Conv}$ is the heat convection coefficient, and $A$ is the solid surface that is in contact with the void that is filled with air. Therefore, at the free surfaces of the solid materials $\Omega_{s}$, the convection is taking place, so the element on the solid’s boundary is needed to be updated during the optimization process using the term ${\mathbf{K}}_{Conv}^{i}$ such that:

5 $$ {\mathbf{K}}_{Conv}^{i} = \int_{{\Omega_{s} }} {{\mathbf{N}}^{T} {\mathbf{Nk}}_{Conv} d\Omega_{s} } $$

where ${\mathbf{N}}$ is the shape function matrix for the heat distribution field on the surface. Moreover, the corresponding heat flux vector load update is formulated as follows:

6 $$ {\mathbf{Q}}_{Conv}^{i} = \int_{{\Omega_{s} }} {{\mathbf{N}}^{T} {\mathbf{k}}_{Conv} {\mathbf{T}}_{\infty } d\Omega_{s} } $$

In this research, the heat conductivity maximization objective function is taking the form:

7 $$ \begin{aligned} & find\quad {\mathbf{x}} \\ & \min :\;C({\mathbf{x}}) = {\mathbf{Q}}^{T} {\mathbf{T}} \\ & s.t.\;\left\{ \begin{gathered} {\mathbf{K}}({\mathbf{x}}){\mathbf{T}} = {\mathbf{Q}} \hfill \\ \int_{\Omega } {{\mathbf{x}}d\Omega } \le v,\;{\mathbf{x}} = 0|1,\;\forall {\mathbf{x}} \in \Omega \hfill \\ \end{gathered} \right. \\ \end{aligned} $$

where ${\mathbf{x}}$ is the binary design variable controlling whether i^th^ element is solid or void ((0) or (1)). $v$ is the volume fraction to be satisfied for the design domain $\Omega$.

In order to extend the applicability of sensitivity analysis for multiscale analysis, we've expressed all equations using Newton notation to depict the derivatives.

The derivative of heat compliance (i.e., $\dot{C}$) is taking the general form as:

8 $$ \dot{C} = {\dot{\mathbf{Q}}}^{T} {\mathbf{T}} + {\mathbf{Q}}^{T} {\dot{\mathbf{T}}} $$

where ${\dot{\mathbf{Q}}}$, ${\dot{\mathbf{T}}}$, are the first order derivative of the heat load and temperature with considering Newton notation. ${\dot{\mathbf{Q}}}$ can be obtained by deriving equilibrium equation for heat conduction, such that:

9 $$ {\dot{\mathbf{Q}}} = {\dot{\mathbf{K}}\mathbf{T}} + {\mathbf{K}} {\dot{\mathbf{T}}} $$

So, the term ${\dot{\mathbf{T}}}$ can be expressed as:

10 $$ {\dot{\mathbf{T}}} = {\mathbf{K}}^{ - 1} \left( {{\dot{\mathbf{Q}}} - {\dot{\mathbf{K}}\mathbf{T}}} \right) $$

Consequently, the sensitivity for a general compliance minimization problem can be formulated as follows:

11 $$ {\mathbf{C}} = 2{\dot{\mathbf{Q}}}^{T} {\mathbf{T}} - {\mathbf{T}}^{T} {\dot{\mathbf{K}}\mathbf{T}} $$

In case of fixed loading condition, the first term of Eq. (19) (i.e.,${\dot{\mathbf{Q}}}$) will vanish leaving the sensitivity to become:

12 $$ \dot{C} = - {\mathbf{T}}^{T} {\dot{\mathbf{K}}\mathbf{T}} $$

It is apparent from Eq. (12) that the minimization of heat compliance is exhibiting the characteristics of a self-adjoint problem.

### EMI objective function

**Figure 1:**
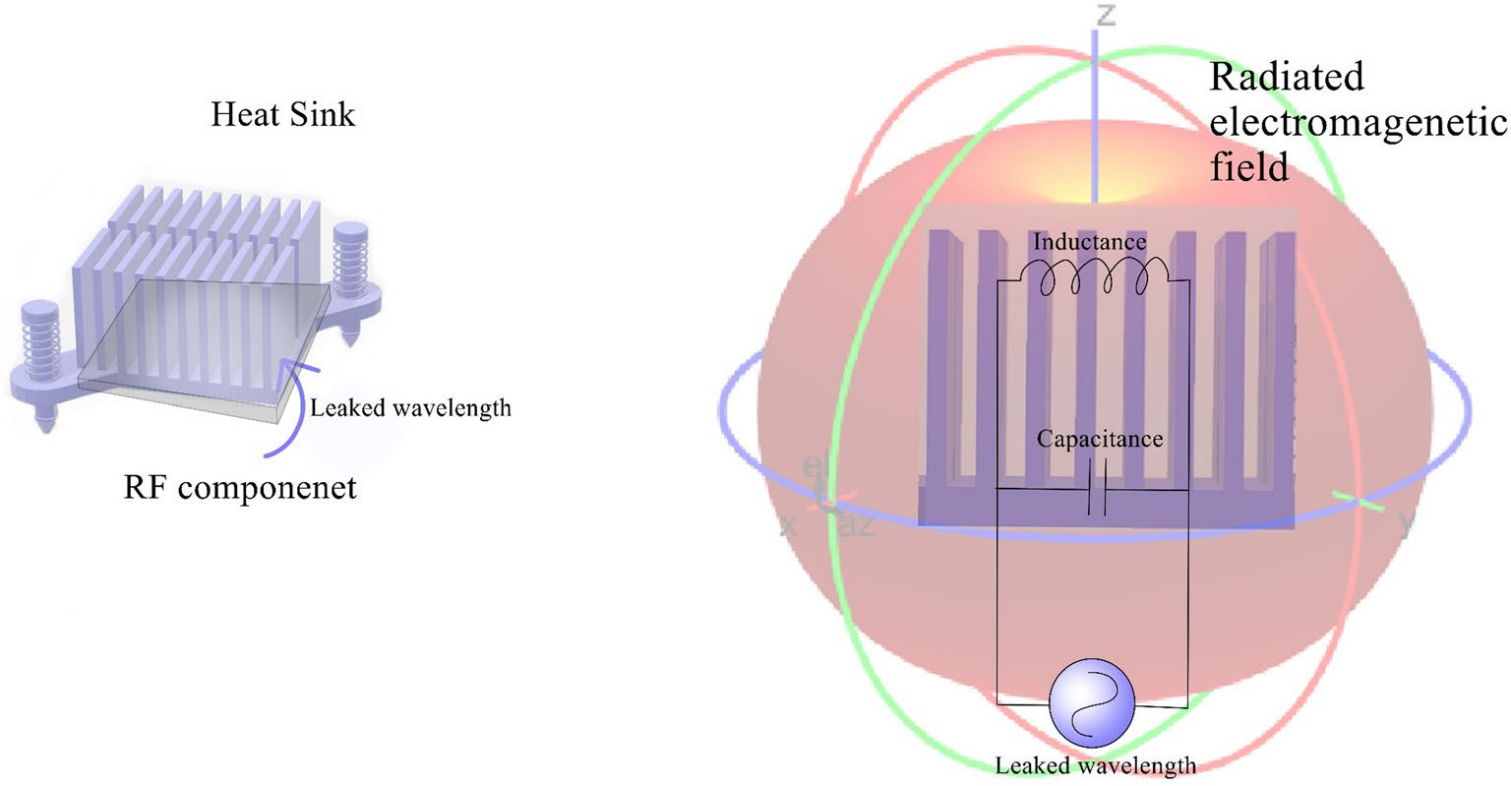
The equivalent electromagnetic circuit of heat sink.

EMI is electromagnetic disturbance from devices, disrupting function and possibly exceeding human exposure limits for specific absorption rate (SAR). SAR measures human body energy absorption from electromagnetic fields, like mobile phones. EMI is simulated using waveguide propagation and frequency behavior. Engineers refer to the source as RF circuit with fixed wavelength. In simulations, the metallic structure (e.g., heat sink) serves as principal RF circuit (Fig. 1). When leaked frequency aligns with heat sink inductance and air capacitance, it becomes an unintended antenna. Evaluating its emissivity/absorptivity is vital. Antenna wave propagation is intricate due to factors like medium conductivity, obstacles, reflections, and wave traits. Precise electromagnetic wave measurement poses challenges. Near-field and far-field scanning, simulations, common methods, have limitations in capturing wave behavior.

When the leaked frequency aligns with the resonance frequencies of the metallic heat sink's inductance and the surrounding air's parasitic capacitance, the heat sink may inadvertently function as an antenna. In such cases, it becomes crucial to assess the metallic structure's emissivity/absorptivity for electromagnetic waves. Comprehending wave propagation from an antenna entails considering various factors, including medium conductivity, obstacles, reflections, and wave characteristics. However, accurately measuring electromagnetic waves poses challenges, with techniques like near-field and far-field scanning and simulations commonly employed, albeit each with its limitations in capturing wave behavior. To facilitate electromagnetic wave analysis within and beyond the metallic object, researchers have introduced various parameters, including admittance, impedance, scattering transfer, scattering, and hybrid parameters. These parameters play crucial roles in RF component design and evaluation, with a notable emphasis on scattering parameters ($S_{ij}$)^36^. $S_{ij}$ have emerged as the most popular and widely utilized parameters for waveguide RF assessment, owing to their effectiveness in characterizing the behavior of electromagnetic waves within the metallic structure and their interaction with surrounding elements. In RF circuits, it is often described using wave quantities which are the incident wave (Wx) and the reflected wave (Wr) within the RF circuit within the Euler operator ($e^{i\theta }$).

13 $$ {\text{Wx}} = E_{input} e^{i\theta } $$

14 $$ {\text{Wr}} = E_{reflected} e^{i\theta } $$

where $E_{input}$ and $E_{reflected}$ are the electric potential of the incident and reflected wave. The ratio of the reflected wave is the complex reflection coefficient (Γ), which denominates the relation between the reflected and the incident waves:

15 $$ \Gamma = \frac{Wr}{{Wx}} $$

Therefore, by connecting the device under testing (DUT), to the VNA device, with single port or two ports; it is easy to evaluate the ability of DUT to omit the targeted RF practically. Moreover, using numerical method for evaluating $S_{ij}$ is highly recommended.

Numerically, the scattering parameters are evaluated using finite element as the following:

16 $$ {\mathbf{S}}_{ij} = \frac{1}{2}{\mathbf{E}}^{T} {\mathbf{K}}_{A} {\mathbf{E}} $$

where ${\mathbf{E}}$ is the electric filed, and ${\mathbf{K}}_{A}$ is the global antenna finite-element electromagnetic stiffness matrix. Reducing the potential for the optimized topology heat sink to act as an antenna for leaked electromagnetic power has been achieved through EMI minimization efforts. This is accomplished by evaluating the Voltage Standing Wave Ratio (VSWR)^37^. VSWR is taking the form:

17 $$ VSWR = \frac{{1 + \left( {\left| {E_{input} } \right|/\left| {E_{reflected} } \right|} \right)e^{i\theta } }}{{1 - \left( {\left| {E_{input} } \right|/\left| {E_{reflected} } \right|} \right)e^{i\theta } }} $$

where $\left| {E_{input} } \right|$ is the absolute supplied electric potential on the antenna, $\left| {E_{reflected} } \right|$ is the reflected electric potential to the source, and $\theta$ is the phase difference between the applied and reflected electric potentials. As such, the VSWR can be rewritten in terms of the $S_{ij}$ as:

18 $$ VSWR = \frac{{1 + |S_{11} |}}{{1 - |S_{11} |}} $$

### Realizing high heat conductivity with low EMI structure using the MASTO method

MASTO improves topology optimization's efficiency, speed, and quality. It combines YUKI's hybrid optimization and gradient descent. MASTO defines binary design area by YUKI. It evaluates and splits area into local and exploration regions. It divides variables into sets without adaptive functions. It increases global solutions^38,39^.

Various optimization methods are employed to attain optimal solutions and minimize suboptimal outcomes. In topology optimization, vital for sizable problems, heuristic methods are crucial. They trim computational costs and enhance optimization stability. Yet, optimal design hinges on factors like search increment choices and initial algorithm point. The latter is vital, especially in intricate functions like maximizing heat conductivity in point-to-volume approaches^14,34,40^. Metaheuristic algorithms excel in general-purpose optimization, not requiring gradients for practical deployment. Their multipoint search distinguishes them from gradient descent, enabling extensive solution space exploration and global extrema identification. Metaheuristics' multipoint strategy benefits initial point selection in topology optimization, yielding optimal designs with reduced computational costs^34^. In this study, our aim is to attain multiphysics optimization of structures that exhibit both elevated heat conductivity and minimal EMI concurrently. To achieve this dual objective, we adopt an optimization methodology centred on the reduction of the ratio between heat resistance and the VSWR inherent in the design configuration, as illustrated in Eq. (19).

19 $$ \begin{aligned} & find\;{\mathbf{x}} = [{\text{x}}_{i = 1..N} ] \\ & \min :\;f\left( {\mathbf{x}} \right) = \frac{C}{VSWR} \\ & {\text{s}} .t.\;\left\{ {\int\limits_{\Omega }^{{}} {{\mathbf{x}}d\Omega } \le V,\;{\mathbf{x}} = 0|1\forall {\mathbf{x}} \in \Omega } \right. \\ \end{aligned} $$

**Figure 2:**
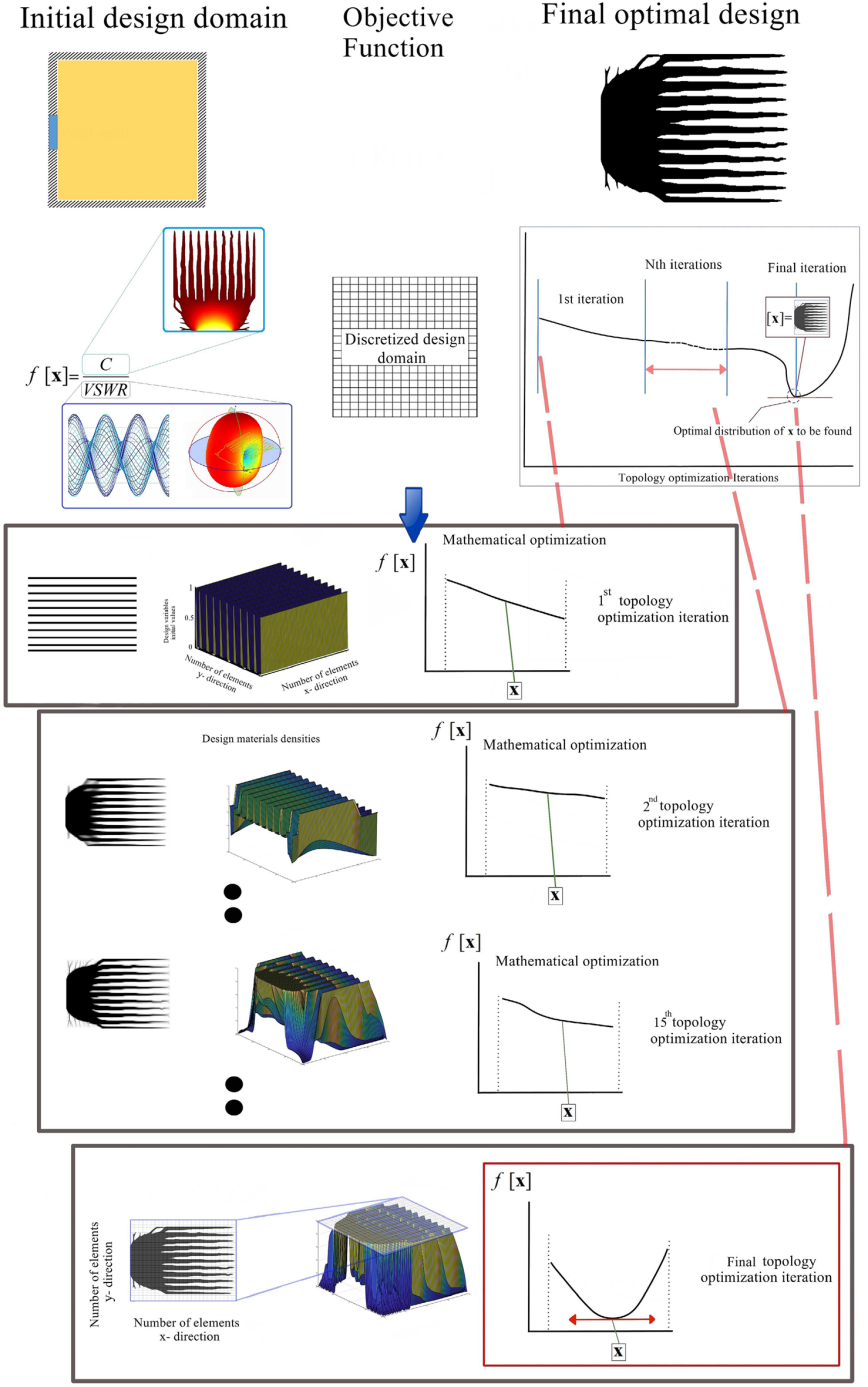
Optimization process with MASTO.

**Figure 3:**
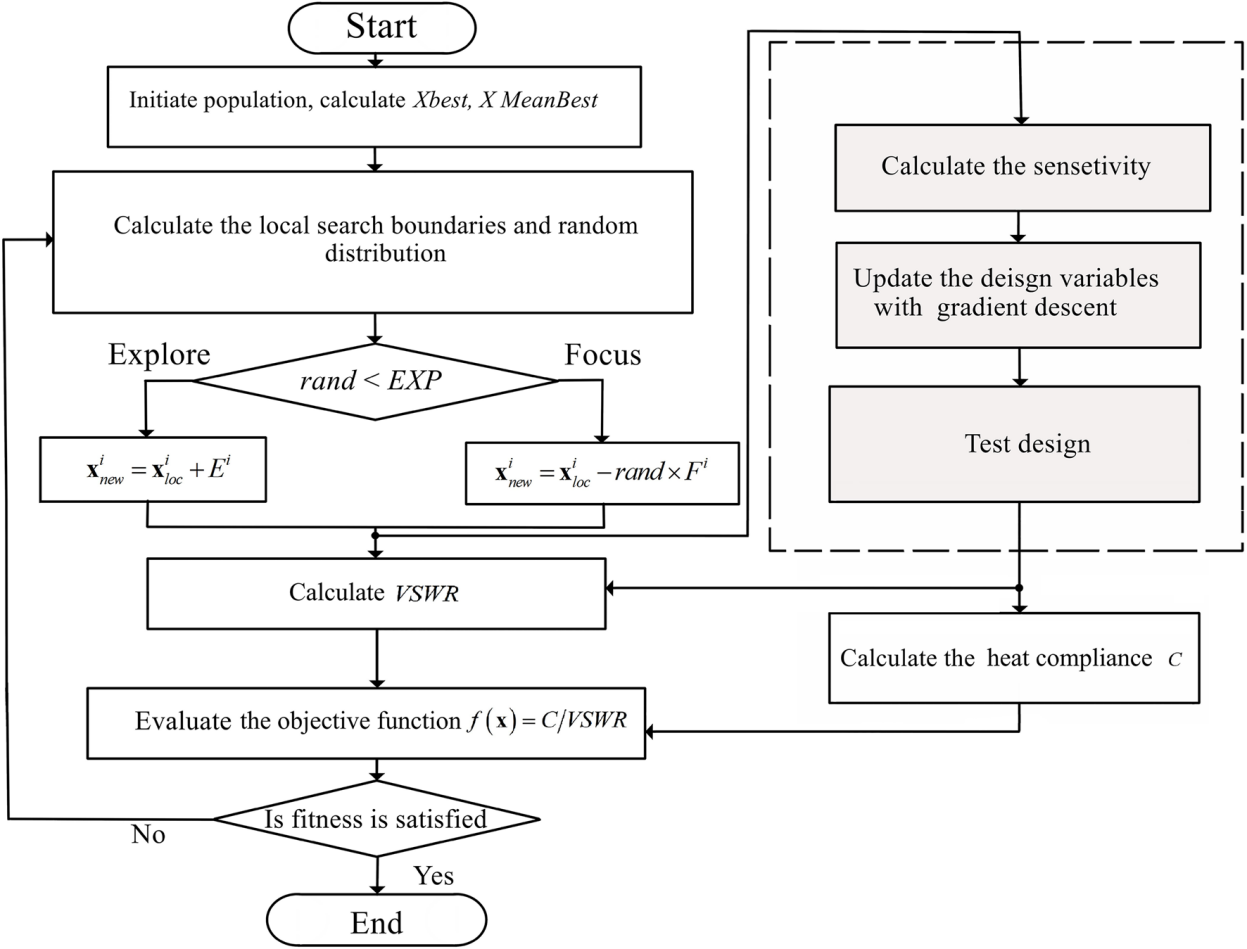
The flowchart of MASTO optimization method.

To enhance the efficiency of topology optimization process, we employ a method that explores multiple initial search points^34,41^. MASTO optimization method starts with initializing candidate solutions from the YUKI algorithm, forming initial strip lines in the design domain (Fig. 2). YUKI explores solution spaces to balance exploration—searching diverse regions—and exploitation, refining solutions in promising areas. This blend prevents local optima traps and improves solutions near high-potential regions. Synergizing these approaches expedites the search for structures with desired attributes like exceptional heat conductivity and minimal EMI. Optimization employs in-house MATLAB codes integrating partial differential equation and antenna toolboxes. MASTO maximizes a predefined objective function, focusing on the heat compliance to Voltage Standing Wave Ratio (VSWR) ratio. Its framework (Fig. 3) begins with population generation, computing optimal and mean design variables. Local search boundaries are set, defining variable ranges, using stochastic processes for exploration or focus. Exploration introduces random perturbations within boundaries to generate new solutions, while focus adjusts variables towards optima. Iterative processes continue until convergence, driven by fitness criteria evaluation. Sensitivity analysis measures responses to variable modifications. Gradient descent updates variables iteratively, aligning them with the steepest objective function descent. Heat compliance assessment tests design resilience iteratively until algorithmic completion. Supplementary materials provide additional details on the MASTO method.

## Numerical investigation

**Figure 4:**
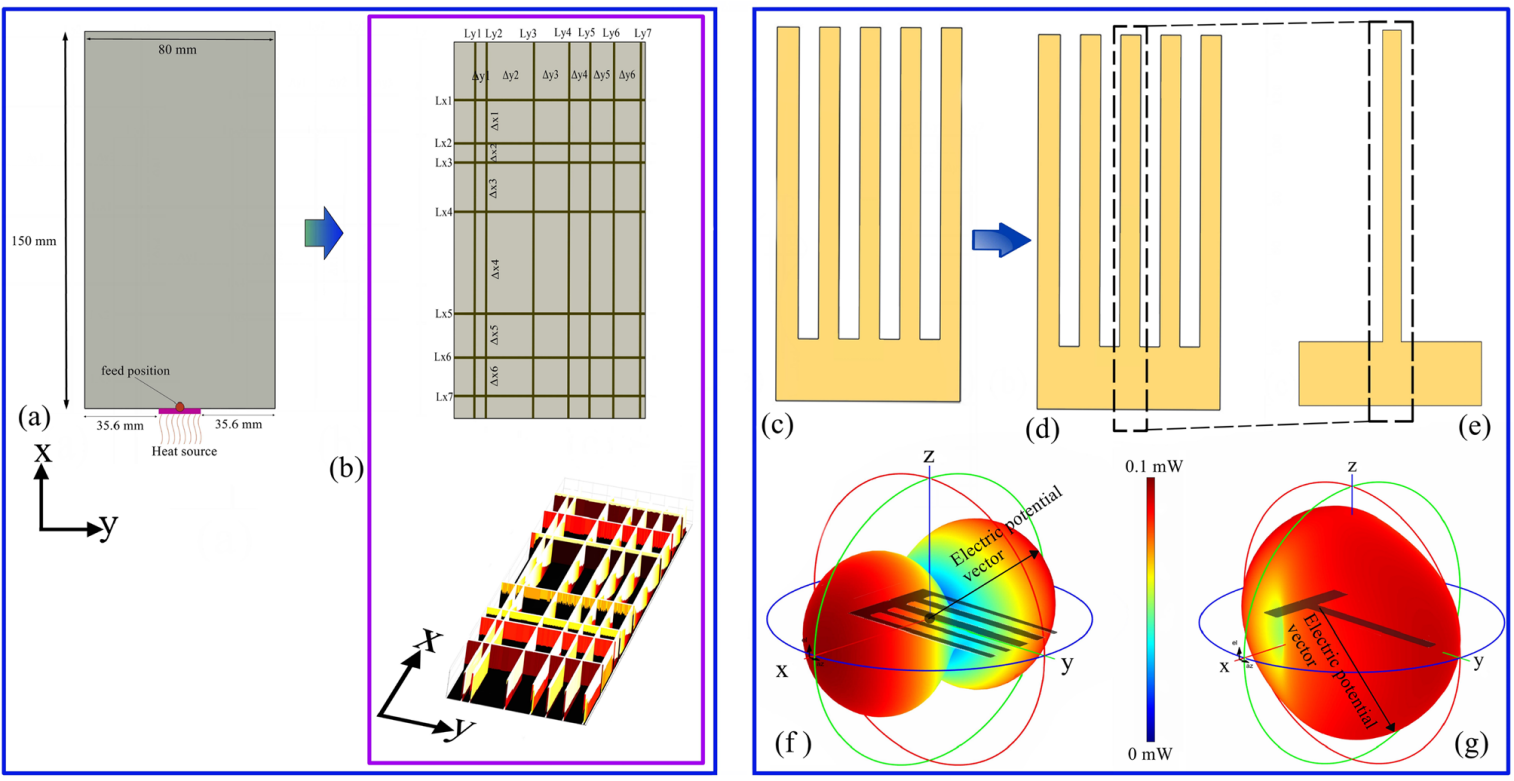
The spatial configuration includes (**a**) the design domain, (**b**) representation of the initial guidance configuration for the MASTO method, (**c**–**e**) the initial test designs, and (**f**, **g**) the polar representation of the electric potential vector.

As an integral component of the MASTO framework, this section focuses on layout optimization of structure with high heat conductivity and low electromagnetic interference potential. The primary objective here is to simultaneously address the maximization of heat transfer efficiency and the reduction of EMI effects. The spatial arrangement of all exemplary cases maintains a consistent dimension of 80 by 150 mm along the x and y directions. In this work, copper is utilized extensively due to its exceptional thermal conductivity of 400 W/mK, making it a preferred material for heat sink core design. Notably, the positioning of critical elements is standardized: the electromagnetic feed, serving as the source of leaked signals, is situated at the center of the lower boundary of the design domain. Similarly, the heat source is also positioned along the lower boundary, as visually indicated in Fig. 4. The research is particularly oriented towards achieving design for low EMI targeted frequency of 850 MHz leakage frequency.

In general, to increase the heat sink performance, the long and parallel radial spiky fins are preferable^14,42^. However, there is a notable risk of these fins resonating with leaked frequencies, potentially becoming a source of electromagnetic interference (EMI). To explore this phenomenon, a 2D design featuring elongated and regular fin-like structures (as depicted in Fig. 4c) was employed to investigate EMI associated with the leaked frequency, utilizing copper as the material. Simulation results indicate a Voltage Standing Wave Ratio (VSWR) of 2.13 for this design. The heat sink radiation polar plot is illustrated in Fig. 4f.

Our analysis suggests that the effective impedance matching in the heat sink design primarily stems from the elongated fin's rectangular structure. To validate this inference, simulations were conducted on a single fin design (Fig. 4e) under analogous conditions. The simulation yielded a VSWR value of 2, emphasizing the structural impact. The radiation pattern of the single fin structure is distinct, as depicted in Fig. 4g. Despite discernible dissimilarities in the spatial configuration of metallic structures, the VSWR remains consistent between the two cases.

The subsequent phase involves employing the Metaheuristic Aided Structural Topology Optimization (MASTO) method to configure a heat sink design emphasizing efficient heat dissipation and minimal electromagnetic interference (EMI). The exceptional efficacy of MASTO stems from its adept utilization of the initial design domain, facilitating multi-point searches across diverse search spaces. This capacity enables MASTO to intricately explore design possibilities, significantly enhancing the likelihood of attaining a solution excelling in both heat dissipation and EMI mitigation. The dynamic utilization of the initial design domain underscores MASTO's proficiency in employing diverse search strategies for optimal outcomes in complex optimization scenarios. To achieve this dual objective, three distinct design criteria were meticulously chosen. In the first criterion, an initial design domain comprising seven parallel strip lines, each with a width of 1 mm, was introduced to the x and y directions, cumulatively establishing 14 lines within the design domain.

The visual representation of these initial design domains is provided in Fig. 4b. It's important to note that the spacings between the lines in both the x and y directions, denoted as parameters $\Delta {\text{x}} (1...{\text{N}} )$ and $\Delta {\text{y}} (1...{\text{N}} )$, are deliberately assigned random distributions within the confines of the YUKI algorithm design spaces. This strategic incorporation of variability in spacing enhances the exploration of the optimization landscape.

**Figure 5:**
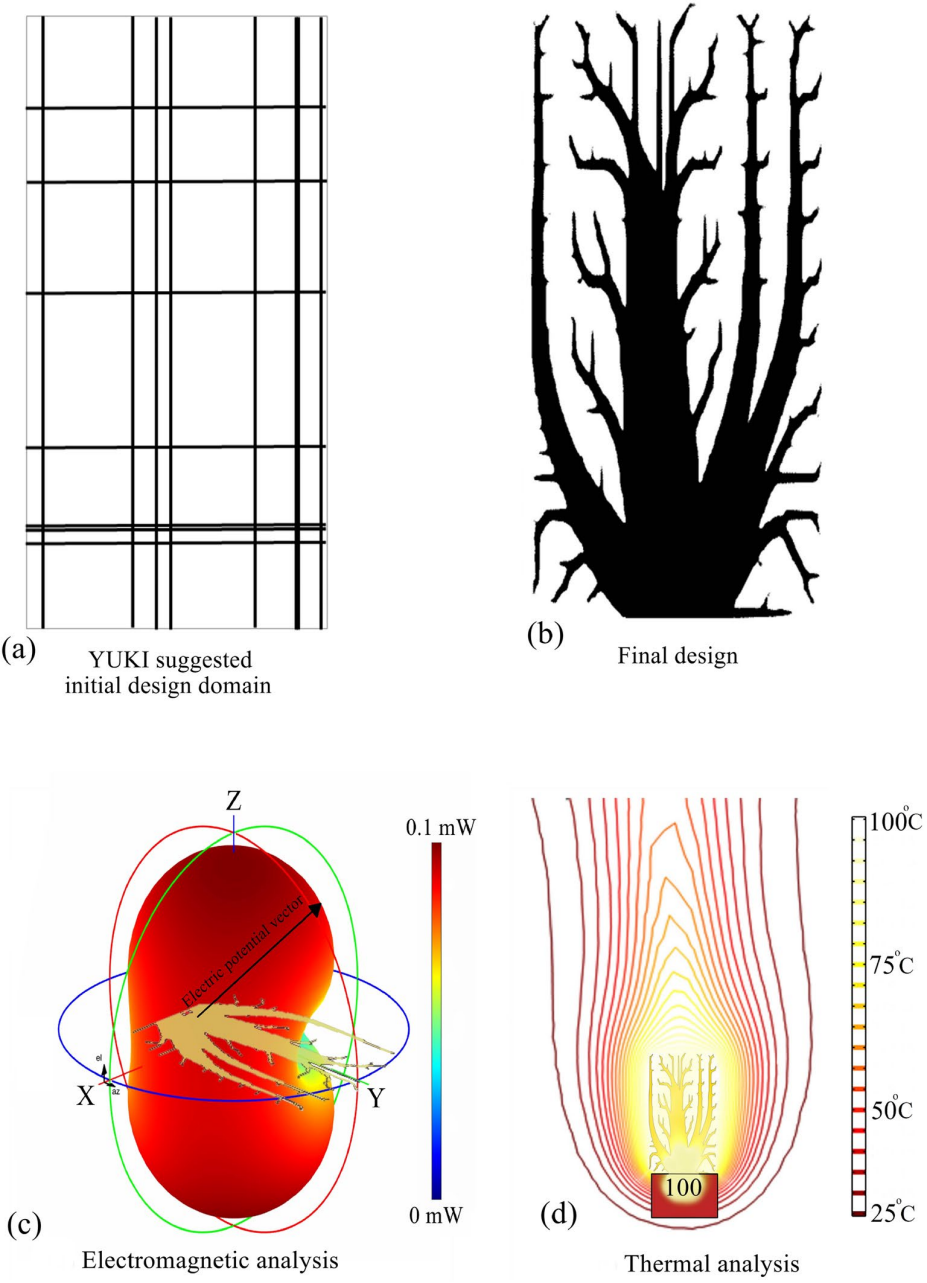
Conceptual initial design domain and the results of the 14-line optimization case, featuring: (**a**) representation of the initial guidance configuration for the MASTO method, (**b**) the final design, (**c**) the polar configuration of the electric potential vector, and (**d**) the thermal analysis.

**Figure 6:**
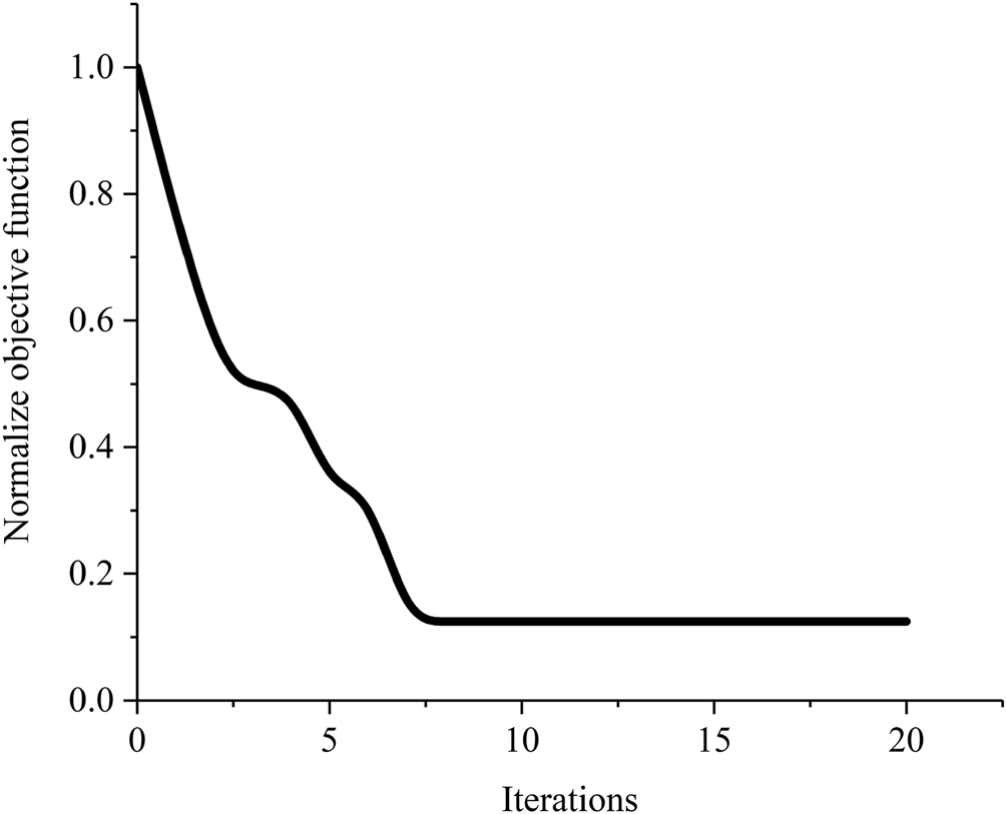
The objective function history of MASTO method for 14 lines initial design domain.

In this pursuit, an optimization task was undertaken with a population size of 20, allowing for the efficient evaluation and refinement of potential solutions. The culmination of this process is evidenced by the presentation of optimal designs in Fig. 5. The simulation results for the heat performance and electromagnetic radiation of the design case with 14 initial lines are depicted in Fig. 5c,d respectively. The objective function history is presented in Fig. 6.

The VSWR for the resulted design is 55.62, which is higher enough to consider attaining successful design in term of not being good source of the leaked electromagnetic signal, and i.e., having low electromagnetic interference.

In our exploration of the design methodology and performance assessment across multiple design scenarios, we investigated a second and third design criteria with thorough precision. For the second criterion, we employed a configuration featuring five initial strips, amounting to a total of 10 lines. In the case of the third criterion, we deliberately introduced three initial strips, resulting in a combined total of 6 lines. Our approach aimed to ensure a thoughtful and intentional incorporation of each strip. The resulting optimal designs, as well as the most effective initial configurations for the 14-line, 10-line, and 6-line designs, are comprehensively presented in Table 1.

**Table 1:**
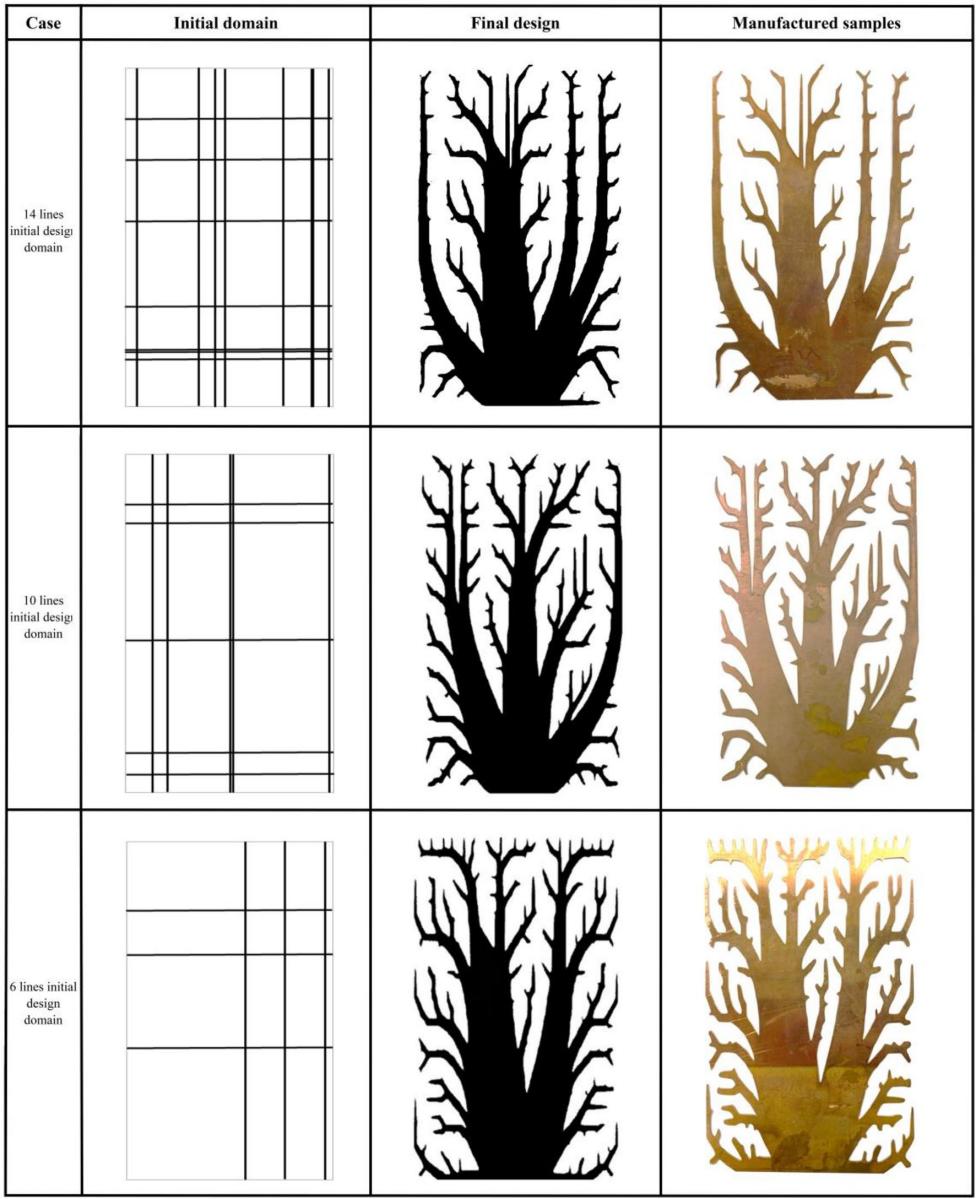
The initial design domains of 14, 10, and 6 lines, and the final MASTO design.

**Figure 7:**
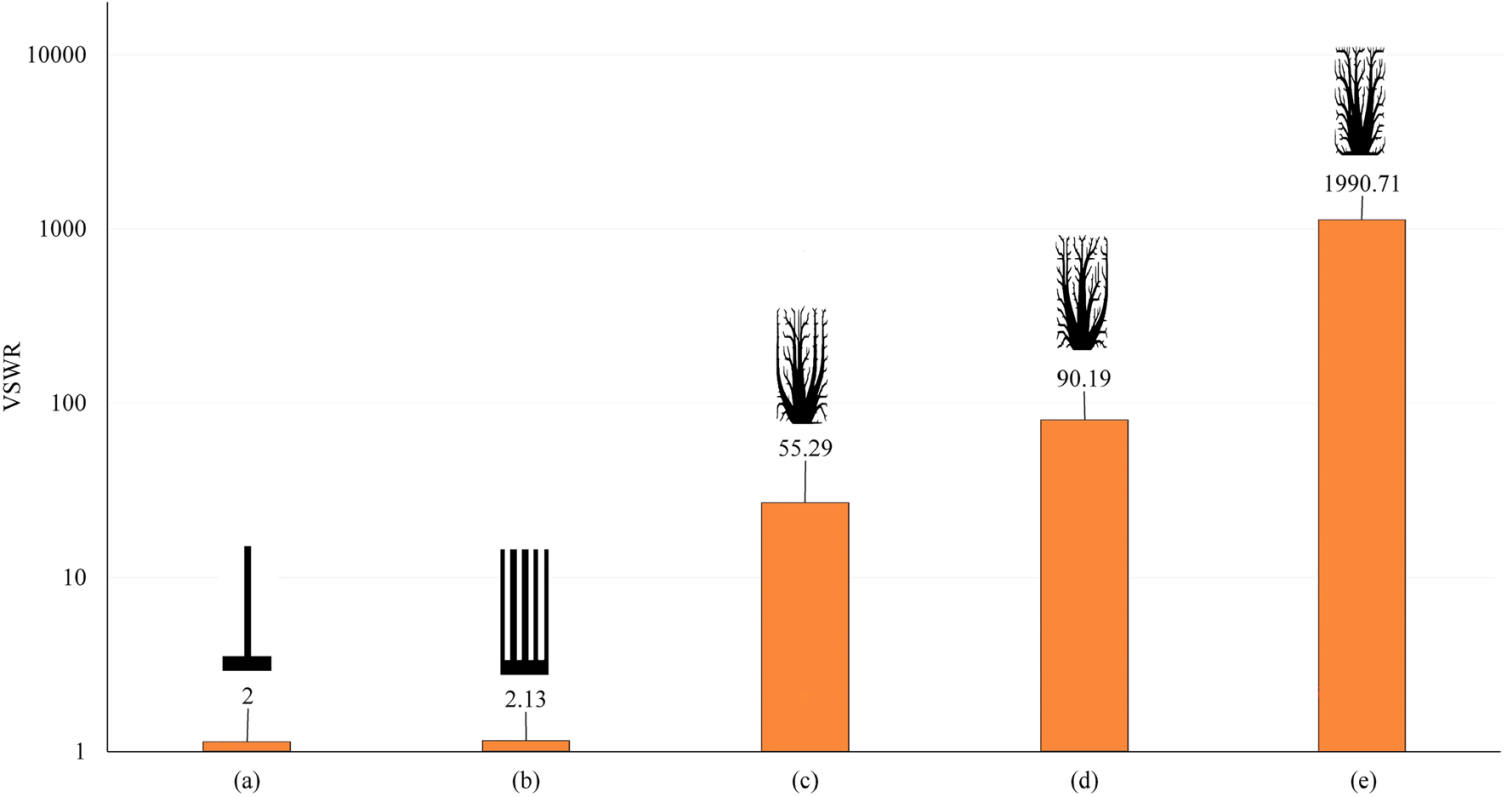
Numerical results of the VSWR for (**a**) Single spike control design, (**b**) 5 spikes control design, (**c**) the design of 14 lines initial design domain, (**d**) the design of 10 lines initial design domain, and (**e**) the design of 6 lines initial design domain.

VSWR serves as a key metric to assess leaked frequency containment and radiative emission reduction in the mentioned designs. VSWR analysis strongly supports radiative mitigation effectiveness. Non-optimized Single Spike Control's VSWR is 2, 5 Spikes Control remains at 2.13. The 14-line initial design domain, optimized through MASTO, achieves VSWR of 55.29, showing notable EMI reduction. Similarly, optimization of 10-line initial design domain yields a remarkable peak VSWR of 90, indicating higher electromagnetic integrity. Optimizing 6-line initial design domain leads to elevated VSWR of 1990.71, underlining intricate design-radiative balance. This thorough analysis underscores MASTO's robust effectiveness in significantly mitigating electromagnetic radiation leakage. It exemplifies potential to limit radiative emissions across diverse design paradigms (Fig. 7).

**Figure 8:**
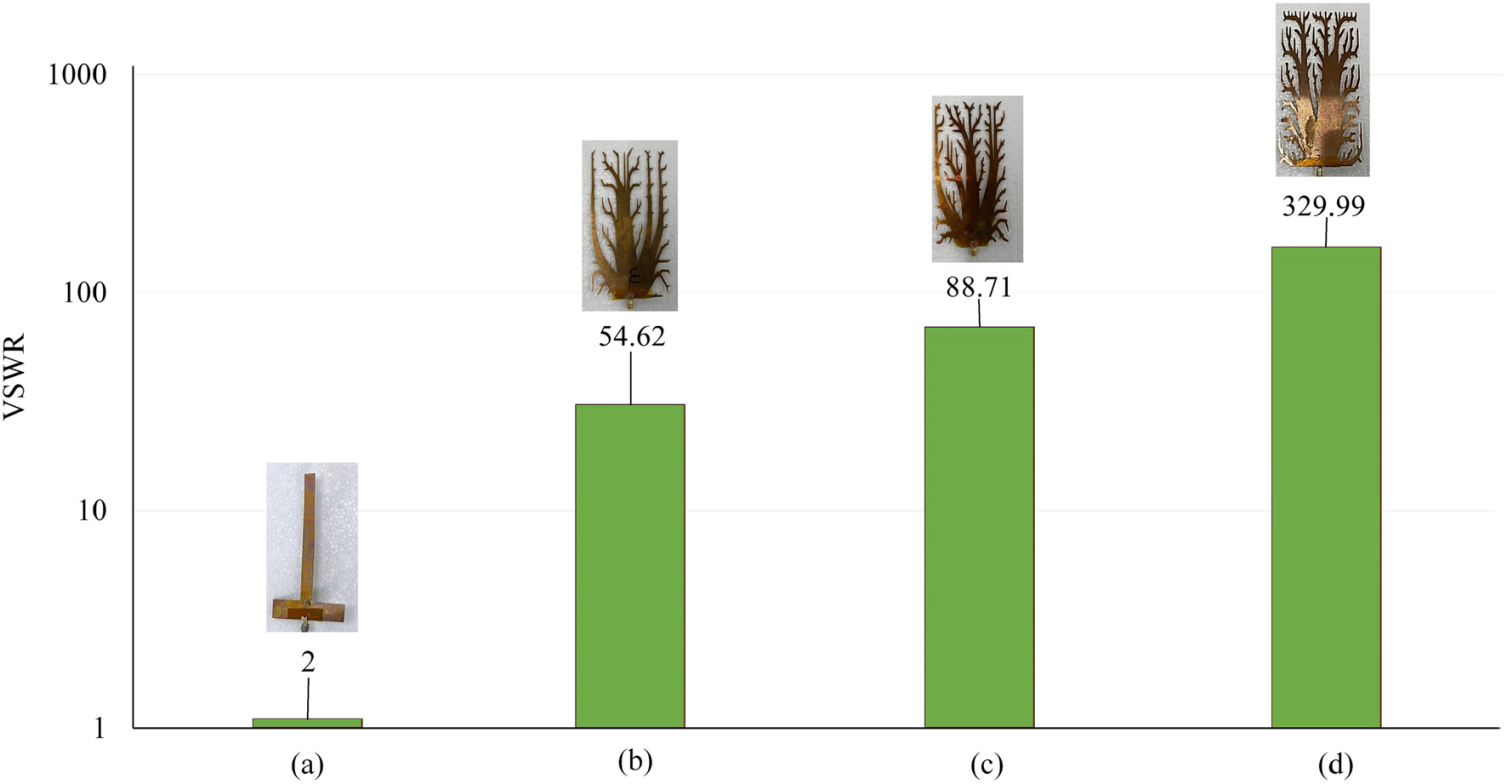
Experimental results of the VSWR for (**a**) Single spike control design, (**b**) the design of 14 lines initial design domain, (**c**) the design of 10 lines initial design domain, and (**d**) the design of 6 lines initial design domain.

To validate results, an extensive experiment assessed synthesized fin design's electromagnetic performance. Copper samples (1 mm thick) were fabricated via electric discharge machining, and equipped with 50 Ω SMA connectors using in house-built infrared reflow soldering machine, positioned from the contact edge to the load (as shown in Fig. 4a). For non-optimized Single Spike Control, VSWR started at 2 (as shown in Fig. 8a). The optimized case of 14-line initial design domain good achieved EMI reduction (VSWR 54.62). The optimized case of 10-line design optimization reached peak VSWR 88.71, indicating a better EMI reduction. The optimized case of 6-line initial design optimization led to higher VSWR (329.99), showcasing complex design-radiative interaction. Analysis confirms MASTO effectively reduces electromagnetic radiation leakage, suppressing emissions across diverse design paradigms (Fig. 8).

**Figure 9:**
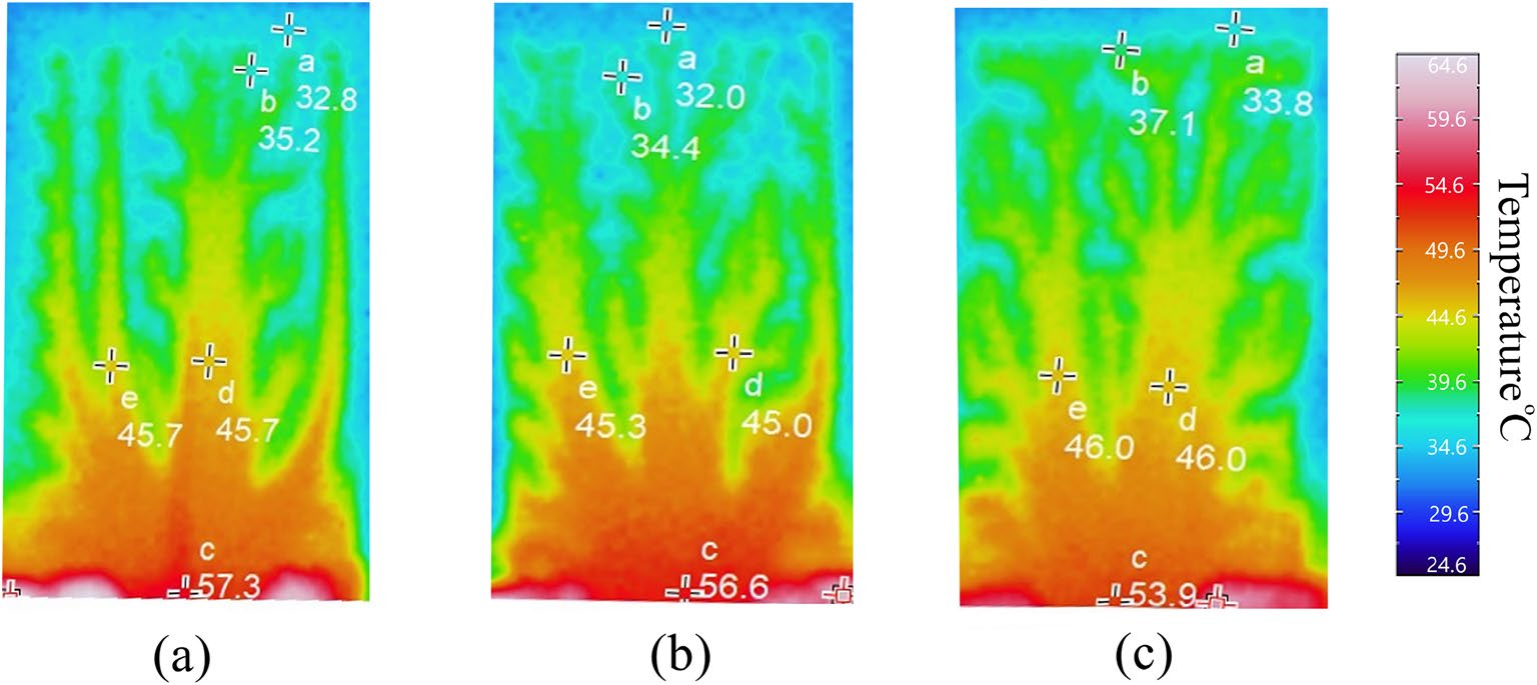
Distributions of the heat sink temperature (Degree centigrade) for the optimized cases of (**a**) 14 initial lines design, (**b**) 10 initial lines designs, and (**c**) 6 initial lines design respectively.

To experimentally validate heat dissipation, a controlled assessment used a piezoelectric heater to subject fin configuration to a fixed thermal load. Thermal camera measurements captured temperature rise. Temperature distributions (°C) for three design cases presented in Table 1, are displayed (Fig. 9). The three designs (14 initial lines design), (10 initial lines design), and (6 initial lines design) were analyzed for their heat conduction and dissipation properties using thermal imaging. (14 initial lines design) exhibited a maximum temperature of 57.3 °C at the base with inefficient heat conduction, evident from steep temperature gradients. Heat dissipation was suboptimal, indicated by cooler upper regions (32.8–35.2 °C). (10 initial lines design) showed improvement, with a maximum temperature of 56.6 °C and more uniform heat distribution (32.0–45.3 °C). However, (6 initial lines design) outperformed both, with a balanced distribution of heat (53.9 °C at the base) and enhanced dissipation (33.8–37 °C), making it the most efficient in both conduction and dissipation processes.

Experimental and theoretical results match, confirming metaheuristic-guided topology optimization. This method creates heat sinks with low interference and high efficiency. Methodology is validated, bridging theory–practice and ensuring accuracy. Optimization agrees with theory, showing consistency, efficacy, and reliability. Agreement demonstrates method's skill in optimizing thermal and electromagnetic aspects, producing multifunctional heat sinks. Consistency highlights value of combining metaheuristics and topology optimization, creating designs that optimize heat and reduce interference. Theory–practice synergy enables advanced designs with superior features.

## Conclusion

This work explores a novel topology optimization using Metaheuristic Aided Structural Topology Optimization (MASTO) to balance high heat conductivity and low EMI potential. The goal was to optimize structures that improve heat transfer and reduce EMI. The study started with examining resonances between leaked frequencies and structures. A 2D design with fin-like structures was tested for electromagnetic properties, identifying designs that worsen EMI by resonance. The investigation used MASTO to find configurations that solve these problems. MASTO performed multi-point searches in different design spaces, showing designs that combine efficient heat dissipation and EMI control. Numerical simulations and experimental validations assessed optimized designs, showing significant EMI reductions, confirming the MASTO optimization. The agreement between theory and experiment proved the methodology's robustness and reliability. A key observation was the structural configuration's role in the designs' performance. The heat dissipation evaluation showed the importance of initial design domain in thermal behaviour. This highlighted the complex relation between design details and thermal management.

### Supplementary Information

Supplementary Information.

## Data Availability

The datasets used and/or analysed during the current study available from the corresponding author on reasonable request.
