# Supplementary material for: Metaheuristic aided structural topology optimization method for heat sink design with low electromagnetic interference
Source: Sci Rep. 2024 Feb 10;14:3431. doi: 10.1038/s41598-024-54083-z (PMC11316020; doi:10.1038/s41598-024-54083-z)
Supplement: Supplementary file 1 — Supplementary Information. [file 41598_2024_54083_MOESM1_ESM.docx]

**Supplementary materials**

In our study, we propose harnessing the crucial role of topology optimization's initial dependency through the application of a metaheuristic approach. This strategic utilization is pivotal for attaining optimal solutions, especially in density-based topology optimization. This dependency emphasizes the significance of the initial design assumption, which serves as the starting point for the optimization process. Through a detailed investigation using the SIMP method, we explored the impact of different initial design domains on the final design and performance outcomes, with a focus on minimizing EMI and maximizing heat conductivity. Our findings revealed that the choice of the initial design domain significantly influences the optimized design and performance. We also introduced a novel approach, the MASTO method, which leverages multiple initial search points, enhancing the efficiency of the optimization process. By combining this method with the YUKI algorithm, we aim to achieve structures that excel in both heat conductivity and EMI reduction, making them versatile for various applications.

1. **Initial dependency of topology optimization investigation**

n this study, we are highlighting the significance of initial dependency concerning the initial design domain in the context of topology optimization. We are also introducing a recommended approach involving the distribution of stripes as an initial design domain. This approach sheds light on the pivotal role of the initial design domain in shaping the early stages of the topology optimization process.

In pursuit of this, we investigated case study (1) with the design variables (x) were initially distributed in a uniform manner across various levels, specifically 1, 0.5, and 0.01. This distribution scheme is graphically depicted in Figures 6(a), 6(b), and 6(c), which also exhibit the corresponding outcomes and optimization iterations. Our investigations were performed for a design domain of 80 by 80 mm the x and y directions. Utilizing a bilinear structured mesh for heat analysis, our overarching goal was to achieve a 50% reduction in weight while concurrently maximizing heat conduction. This was achieved through the application of a uniformly distributed heat load of magnitude 1 across the entirety of the design domain. Additionally, we introduced a heat sink comprising 5 elements along the upper left boundary, while designating the remaining outer boundaries as adiabatic regions. This configuration is visually presented in Fig 1.


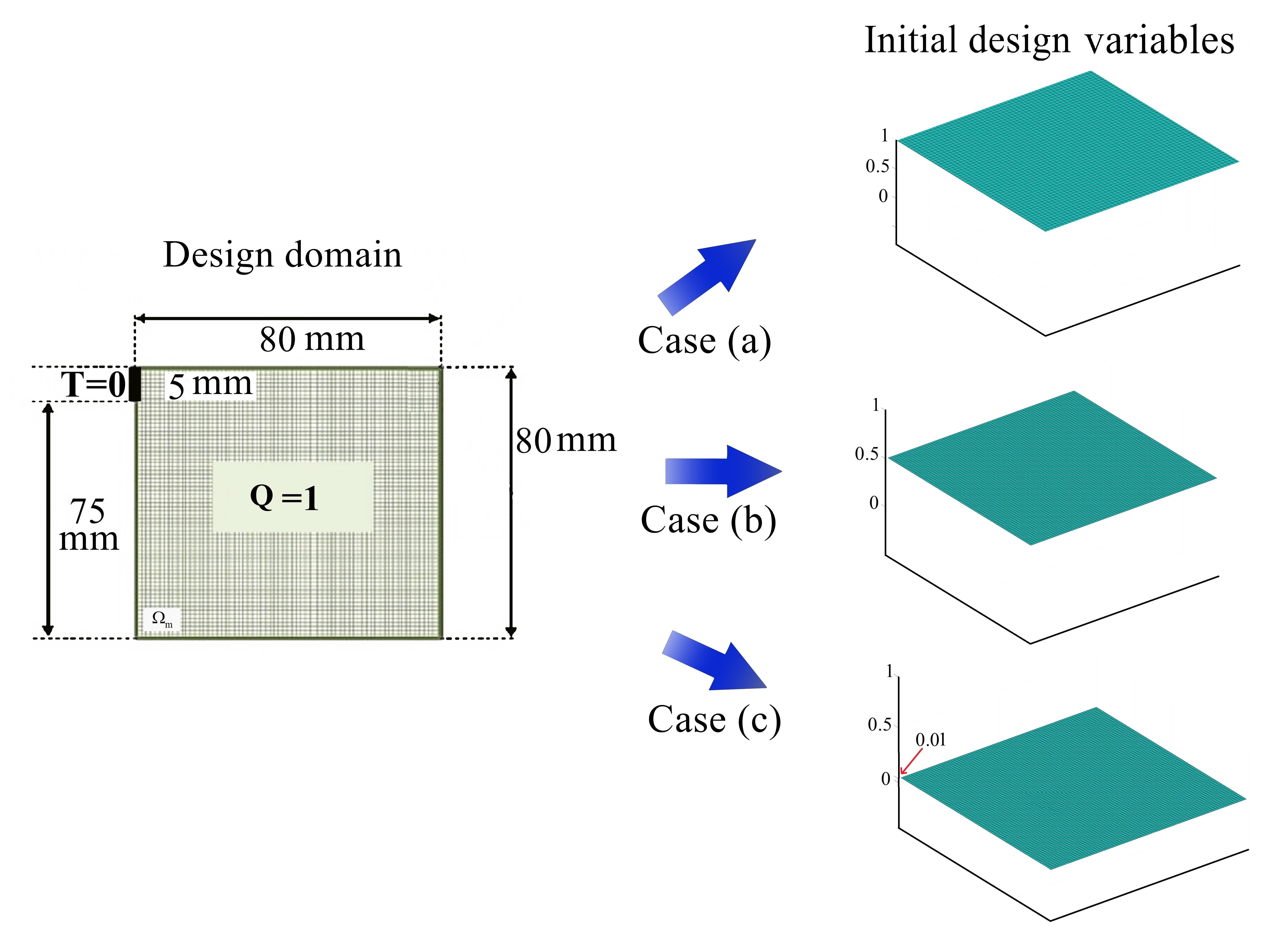


**Fig. 1** Design domains with different levels of the initial levels for design variables.

The optimization procedure comprised a fixed number of 300 iterations. The outcomes resulting from this process, along with the evolution of the optimization iterations, have been meticulously depicted in Figs. 1(a), 1(b), and 1(c), with each figure corresponding to a distinct initial design domain. Additionally, Fig. 2 offers a comprehensive presentation of how different initial design domains influence the performance of the resulting designs. Through a comparative analysis of the observations presented in Figs. 6 and 7, it becomes evident that the selection of the initial design domain plays a pivotal role in shaping both the ultimate optimized design and its subsequent performance.

This investigation highlights discernible disparities in final designs (as evident in Fig. 2) as well as performance (as depicted in Fig. 3). However, it is worth noting that the performance, specifically in terms of minimizing heat compliance, did not exceed a margin of 0.61% (in the case of domain (c) compared to domain (b)). These findings collectively underscore the inherent potential and promise held by the initial design assumption in the topology optimization process. This underscores the essentiality of thoughtfully selecting the starting point, further affirming its role in ensuring the achievement of intended and optimal outcomes.


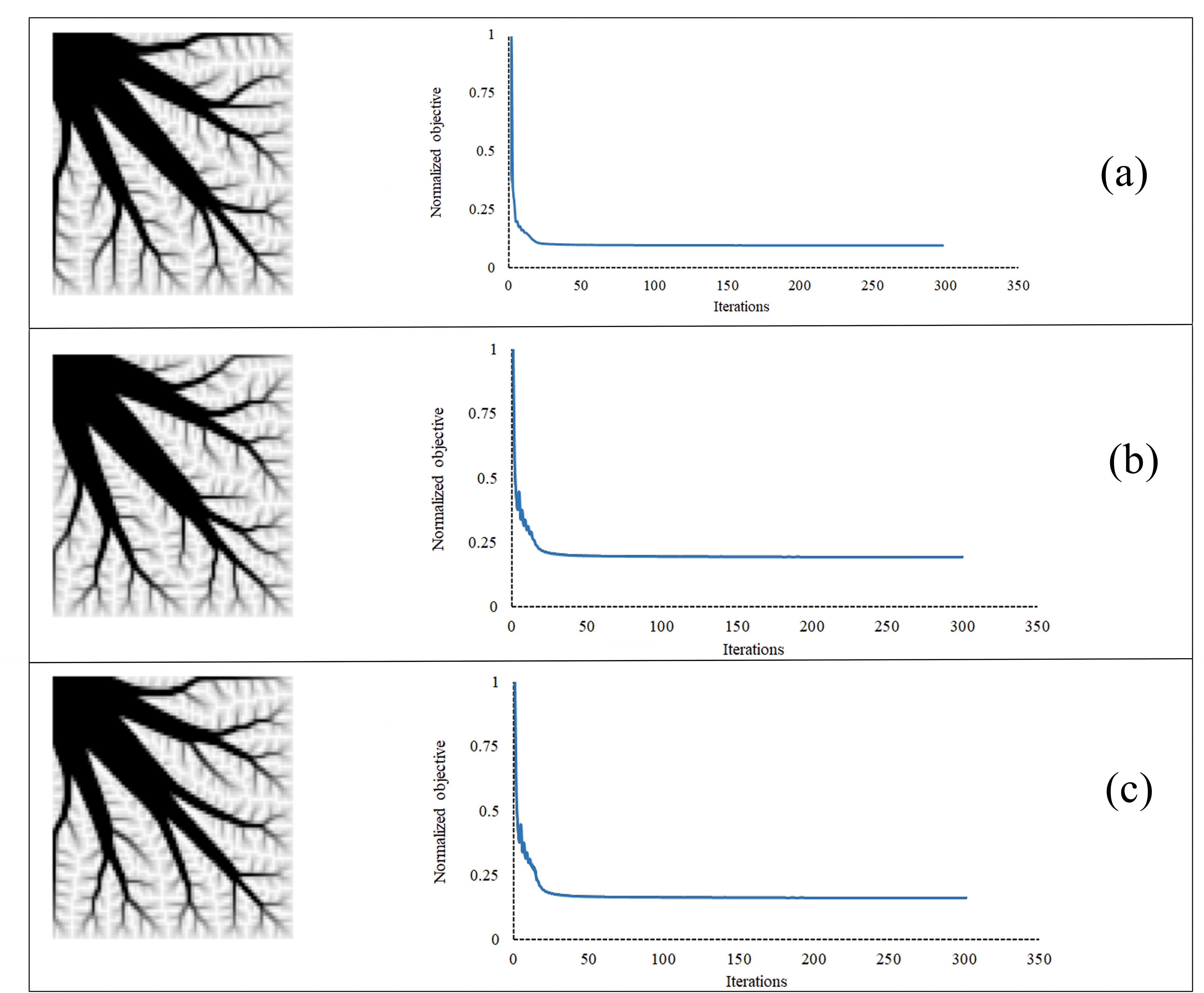


**Fig. 2** Design and the history of the first case study of the initial design domain.


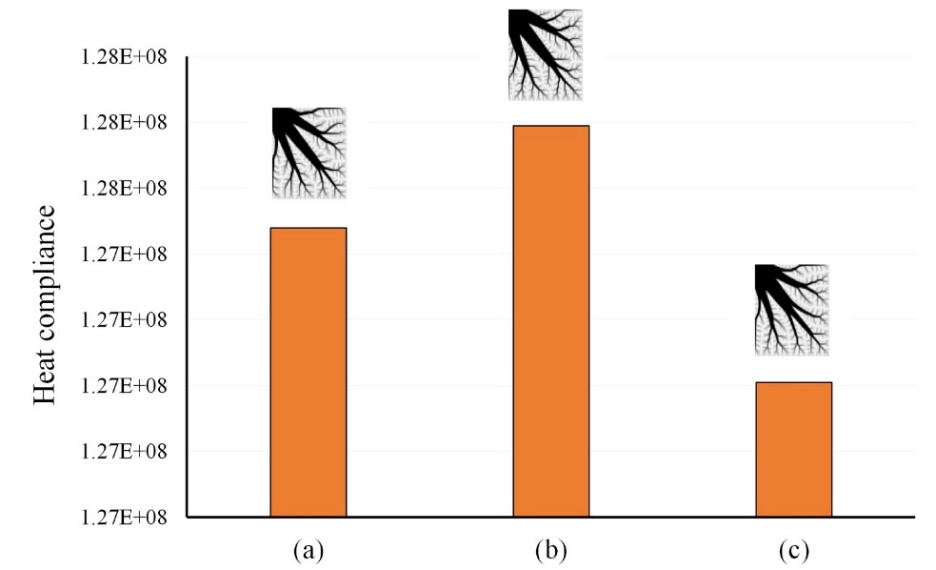


**Fig. 3** Heat compliance of the first study case.

Even though heat compliance proves effective as an objective function for the design of high-conductivity solid problems, the intricacies of design domain discretization demand careful consideration. Equally crucial are the selection of appropriate optimization search steps. These elements are pivotal in the quest to attain an optimal design. Importantly, the success of topology optimization is closely intertwined with the initial design domain, an aspect that researchers have keenly harnessed to enhance the overall performance of topology optimization methods. By strategically manipulating the initial design domain, these studies have demonstrated marked improvements in the optimization process, further underscoring the critical role of this dependency. A detailed discussion of the optimality of heat compliance problem of volume to point and volume to line approach as presented in the paper of Yan et al ^1^. The study undertaken by the authors delved into various scenarios, meticulously analyzing and comparing them, in order to exemplify how an effective design variable interpolation scheme, favorable parametrization conditions, and a discerning selection of the initial design domain can collectively lead to optimal solutions. Drawing insights from Yan et al., it becomes evident that the manipulation of the parametrization factor to influence designs resembling stripes as the initial design domain yields a noteworthy reduction in the optimized heat compliance results. This reduction is in stark contrast to the outcomes achieved when the entire domain is designated as a uniform density field. The impact of presumptions about the initial design domain and the values assigned to it emerges as a notable factor significantly influencing the performance of various topology optimization methods and objective functions ^2345^.

In this study, we propose a novel approach to guide the optimization process away from sub-optimal solutions by initiating the design domain using initial strips. This method is introduced to avoid the complexities associated with incorporating parametrization as a constraint or within the objective function of the topology optimization. Moreover, it aims to facilitate the optimization's convergence to the optimal design without the need for increasing computational demands through elevated design variable counts (such as augmenting mesh resolution ^6^) or diminishing the search step to an extent that decelerates convergence considerably ^1^.

To explore the efficacy of this technique, we employ a design condition akin to case study 1. The design domain spans 200 mm in the x-direction and 80 mm in the y-direction. Consistently, the volume to region approach ^7^. The configuration of the design domain is illustrated in Fig. 4. Diverse initial design domains are tested, encompassing solid, 20-striped, 11-striped, 9-striped, and 5-striped line configurations. Each of these design cases is initiated with an initial design domain level set at 0.5, as depicted in Fig. 4.


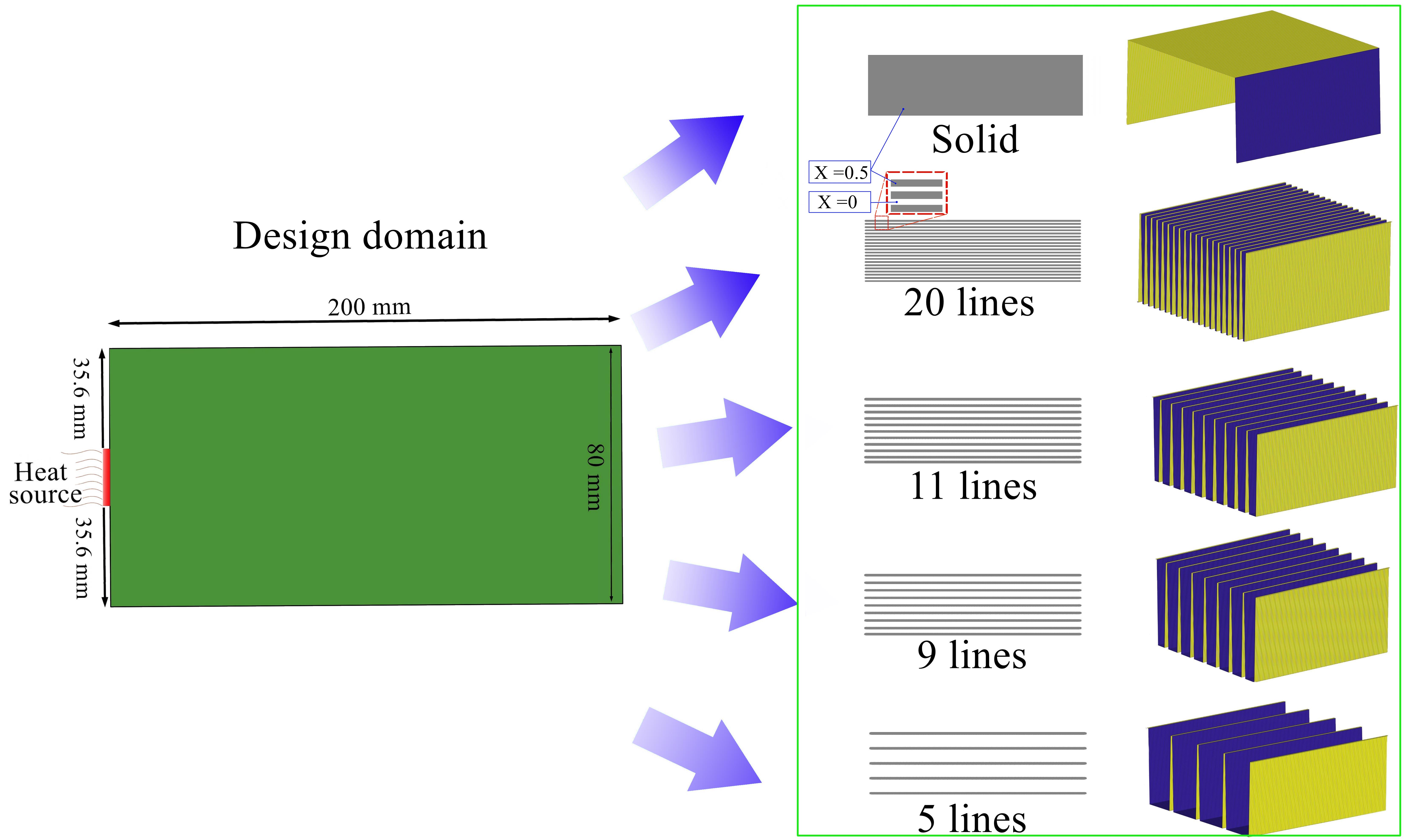


**Fig. 4** Case study 2of the initial design domain effect on topology optimization results.

The designs presented in Fig. 5 exhibit a noteworthy trend of performance enhancement when the design domain is introduced in a linear formation as opposed to a solid bulk configuration. This performance enhancement is clearly evident, with an initial 1% increase in the minimization of heat compliance achieved when utilizing 20 lines as the configuration. Notably, as the number of lines decreases, the performance improvement becomes more pronounced. For instance, in the case of 11 lines, the performance surges by 7.7% in comparison to the solid configuration. Similarly, the configuration with 9 lines demonstrates an 8% performance increase relative to the solid counterpart. Remarkably, the most remarkable performance enhancement in this study is observed in the case of 5 lines, showcasing an impressive 22% increase in performance compared to the solid configuration.





**Fig. 5** Initial values of the design variables versus the performance of topology optimization.

To investigate the influence of the initial design variable on optimization outcomes concerning performance and computational efficiency, a topology optimization study is conducted. This study involves initial design domains composed of five lines, each characterized by different heights ranging from 1 to 0.01, as detailed in the first and second columns of Table 1. The corresponding final designs for each case are exhibited in the third column of the same table, showcasing distinct variations in the final design configurations. Notably, regarding performance, specifically the objective of minimizing heat compliance, the final values demonstrate a striking similarity with negligible deviations. Conversely, in the realm of computational efficiency, a noteworthy trend emerges-higher initial design variable values correspond to expedited convergence towards the final design solution. Furthermore, in contrast to the previous case study where the iteration count was fixed, this investigation adopts a convergence criterion based on the change in the final objective. Termination of the optimization process is triggered by achieving an infinitesimal objective change (1e-3 in this context), signifying the attainment of convergence. The convergence history, elucidated in the fourth column of Table 1, distinctly illustrates a substantial reduction in iteration count for instances where the initial design domain is set to 1, as opposed to cases with lower initial design variable values (0.01 in this specific study). For enhanced clarity and comprehensive illustration of the results, Fig. 6 graphically represents both the iteration count and performance metrics across different levels of initial design domains.

Table 1 Influence of 5 lines initial design variable on optimization outcomes.

**

**





**Fig. 6** Comparative study of performance versus the computational cost of optimization with multiple initial values for the design domain.

1. **Voltage standing wave ratio**

The Voltage Standing Wave Ratio (VSWR) serves as a metric for quantifying the efficiency of radio-frequency power transmission across a transmission line, originating from a power source and propagating towards a load, such as an antenna. In an ideal scenario, where the impedance of the source, the transmission line, and the load are perfectly matched, all energy is efficiently transferred without any reflections. Consequently, the alternating current (AC) voltage remains consistent throughout the transmission line. However, real-world systems often encounter impedance mismatches, leading to partial power reflections back towards the source, akin to an echo effect. These reflections induce destructive interference, resulting in voltage fluctuations along the transmission line. VSWR characterizes these voltage discrepancies by computing the ratio of the maximum voltage amplitude to the minimum along the transmission line. In an ideal setup with no reflections, the VSWR value equals unity (1:1). Conversely, in the presence of reflections, the VSWR value exceeds 1, indicating voltage variations. Mathematically, VSWR can be expressed as the absolute ratio of the maximum ($\left|V_{\max}\right|$) to the minimum ($\left|V_{\min}\right|$) voltage along the transmission line:

$$\mathrm{VSWR}=\frac{\left|V_{\max}\right|}{\left|V_{\min}\right|}$$

Alternatively, it can be derived from the voltage reflection coefficient ($\Gamma$) near the load, obtained from the load impedance ($Z_{L}$) and the source impedance ($Z_{0}$):

$$\mathrm{VSWR}=\frac{1+\Gamma}{1-\Gamma}$$

​ Where $\Gamma$ can be defined as:

$$\Gamma=\frac{Z_{L}+Z_{0}}{Z_{L}-Z_{0}}$$

In instances where the load and transmission line are appropriately matched, the reflection coefficient ($\Gamma$) equals zero, resulting in an ideal VSWR value of 1.0 (1:1). However, achieving a VSWR of precisely 1:1 (or 1.0) in real-world scenarios is challenging due to multifaceted factors intrinsic to the transmission system and its constituent components. These factors, including imperfections in components such as connectors, cables, and antennas stemming from manufacturing processes and material properties, introduce minute impedance disparities that induce signal reflections. Furthermore, environmental influences such as temperature fluctuations, moisture, and electromagnetic interference alter transmission line characteristics and component behaviors, exacerbating impedance variations and signal reflections. Additionally, the length of the transmission line relative to the signal wavelength affects the magnitude and frequency of reflections, even with minor mismatches. Moreover, VSWR exhibits frequency dependence, varying with the frequency-dependent attributes of transmission lines and components, thereby altering impedance matching conditions. Finally, measurement constraints, encompassing inaccuracies in VSWR measurement instruments, calibration discrepancies, cable losses, and signal integrity issues, further exacerbate deviations from the ideal 1:1 VSWR value. It's notable that background noise also plays a significant role in influencing VSWR performance, underscoring the complexity of achieving perfect unity VSWR in practical applications.

In the RF industry, when the VSWR reaches 100:1, it is essentially considered infinite, indicating that the minimum voltage amplitude approaches 0.01 ^8^ (as illustrated in table 2). While VSWR values around (300:1) are possible to be measured, practical measurement for VSAR over (400:1) becomes increasingly difficult due to background noise within the tested device and environmental influences. Nevertheless, in simulations, higher values can be reached due to the absence of consideration for extremely complex noise factors. As VSWR exceeds 100:1, simulations and experimental results begin to diverge significantly, marking a point of total mismatch or infinity. In the theoretical scenario of approaching absolute zero Kelvin, precise measurement of elevated VSWR values encounters hindrances from electronic and atomic vibrations. Metrics such as Return Loss, Reflection Coefficient, and Mismatch Loss elucidate the degree of isolation between the metallic structure and the leaked frequency, offering insights into antenna behavior and resonance characteristics. Additional details can be found in Table 2 for further elucidation ^8^.

Table 2 Conversion Between Return Loss, Reflection Coefficient, VSWR, and Mismatch Loss ^8^.


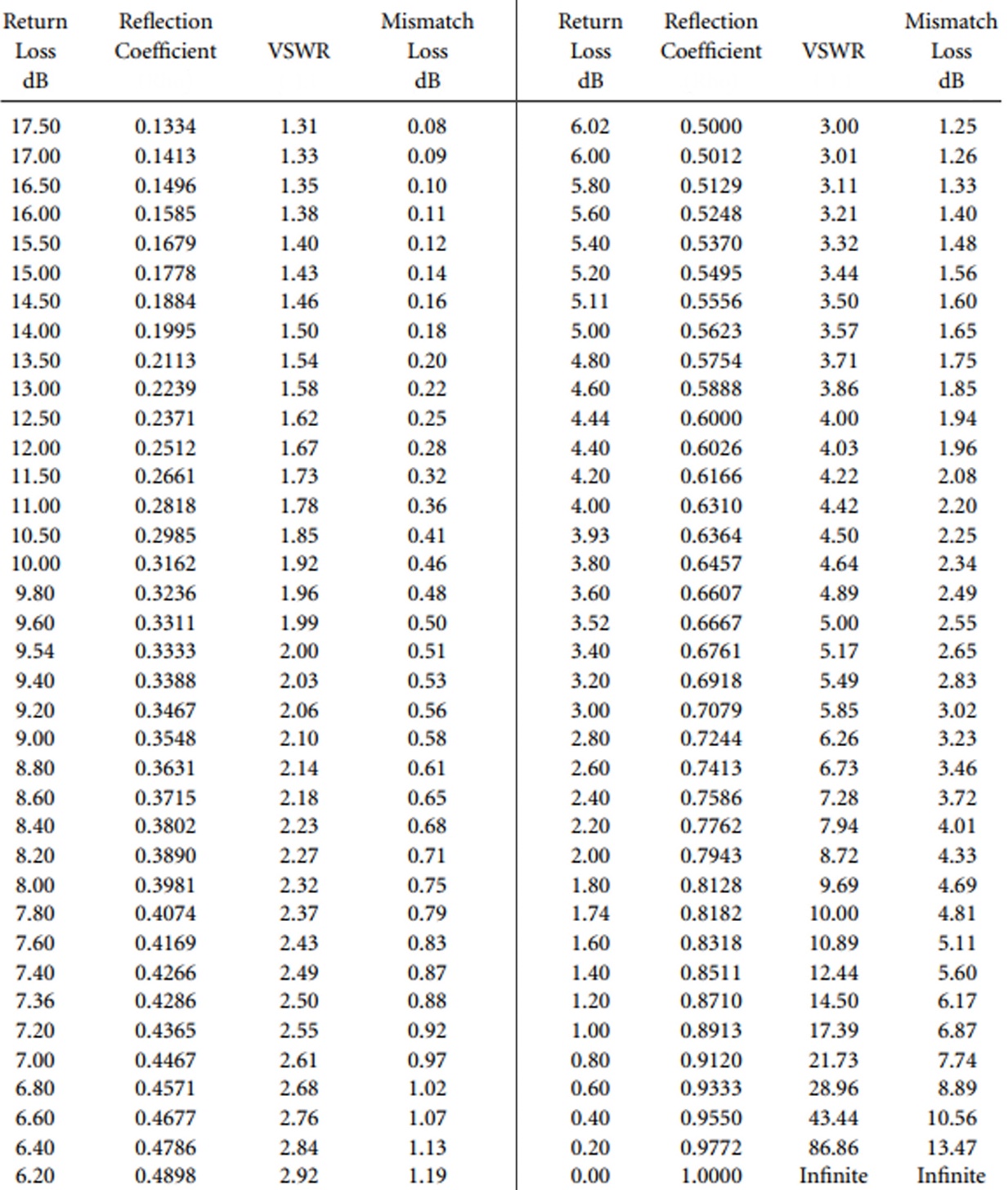


1. **Realizing high heat conductivity with low EMI structure** **using the MASTO method**

Topology optimization's sensitivity to initial values underscores the challenge of achieving global extrema and places a significant burden on the designer's judgment ^91011^. Nevertheless, this sensitivity to initial values, exemplified by the stripes in this study, can be harnessed effectively by robust metaheuristic optimizers. This becomes a notable advantage in the pursuit of conflicting objectives, such as our goals of minimizing EMI and maximizing heat conductivity, all without the need for techniques such as the sum of multi-objective formulations. The primary objective of implementing the MASTO method is to expedite the process of topology optimization while effectively mitigating the risk of getting trapped in local minima. This is achieved by synergistically integrating YUKI's hybrid metaheuristic optimization with gradient descent techniques. To ensure clarity, we will first present a concise overview of the YUKI algorithm formulation, followed by a comprehensive description of the corresponding MASTO formulations.

The YUKI algorithm is meticulously crafted as a metaheuristic optimization technique, adept at yielding optimal solutions with minimal computational burden. This achievement is underpinned by three key characteristics. Firstly, the YUKI optimization algorithm leverages the best solutions obtained in a given iteration ($i$)as a starting point for the exploration of superior solutions in the subsequent iteration ($i+1$).

The YUKI algorithm incorporates the concept of "Best Points," a notion originally introduced in the Particle Swarm Optimization (PSO) method ^1213^ , as the personal best. By capitalizing on the collective knowledge of the competing points, each individual point is enabled to effectively traverse the search space and update its personal best. Consequently, this strategy fosters an advanced global search capability, as the collective knowledge of the swarm continuously evolves and adapts. In each iteration, the population of solutions undergoes evaluation, and a vector is employed to monitor the values corresponding to the best fitness, which, in our case, pertains to the minimized thermal compliance. This best fitness value represents the minimum heat compliance for the specific problem at hand. Subsequently, the Best Points accumulated up to that point are employed to deduce a singular point called the "MeanBest." This MeanBest point acts as a representative of the central position within the cluster of best points, with its value derived from the mean values of these points. The YUKI algorithm initiates by creating a local search area centered around the current best solution achieved thus far. The size of this area is dictated by the distance between this particular point and the MeanBest. This approach embodies several advantageous attributes:

- The local search area diminishes as solutions converge towards the optimum.
- A secure distance is maintained between Best Points to ensure sufficient global search coverage, preventing the local search area from becoming confined to local minima.
- Exploring the local search area can lead to discovering new optimal solutions, dynamically expanding the size of the local search area.
- Over the course of the search, when no new best solution is found, MeanBest is continuously updated, resulting in varied search sizes and a dynamic search process.
- The YUKI algorithm's attributes are contingent on the global search space's magnitude and remain unaffected by individual design variables (search dimensions). Consequently, the local search area's size can significantly differ across dimensions, catering to the distinct sensitivities of each variable. This ensures adaptability to the unique characteristics of every design variable.

The local boundaries are calculated by the following expressions:

$$D=\left|\mathbf{x}_{best}-\mathbf{x}_{MeanBest}\right|$$

$$LB=\mathbf{x}_{best}+D$$

$$LT=\mathbf{x}_{best}-D$$

Where, $\mathbf{x}_{best}$is the variable value corresponding to the absolute best fitness value found so far. $\mathbf{x}_{MeanBest}$is the mean of the “Best Points” vector. $LT$and $LB$are the local upper and lower boundaries of the search space respectively.

The second aspect of the YUKI algorithm involves the division of the search space ($\Omega$). In this study, we partition the population, consisting of the design variables, into two distinct segments. One segment is directed towards exploration beyond the boundaries of the local search area ($\Omega^{EXP}$), while the other segment concentrates on exploring within the confines of the local search area ($\Omega^{Local}$). The size of the population for each segment undergoes a linear adjustment. Initially, the majority of the population is dedicated to exploration, and as iterations progress, an increasing number of solutions shift their focus towards exploring within the local search area. To determine the extent of exploration focality, a randomly generated value between 0 and 1 is compared to the output derived from the following expression ^14^:

$$Evaluate (\mathbf{x}) \left\{\begin{matrix} \mathbf{x}\in\Omega^{EXP}, rand < EXP \\ \mathbf{x}\in\Omega^{Local}, rand > EXP \end{matrix}\right. ,\forall\mathbf{x}\in\Omega$$

Where $rand$ is the random value between 0 and 1, and $EXP$ is the exploration parameter of YUKI algorithm, that assign a percentage of the population to search outside the local search area. Unlike other metaheuristic optimization algorithm, this feature gives the YUKI algorithm the edge in optimizing the computational resources efficiently by focussing on the area that have the best solutions while scanning the other areas at the same time.

The third attribute encompasses an extensive exploration of the focal region through in-depth investigation. This process is initiated by creating an initial random distribution of points within the local search area. Subsequently, the selected points for exploration are employed to calculate novel points positioned beyond the confines of the local search area. The trajectory of exploration is oriented towards regions distanced from the MeanBest, a direction determined by the spatial location of the MeanBest as illustrated in Equation (8).

$$E^{i}=\mathbf{x}_{loc}^{i}-\mathbf{x}_{best}^{i}$$

Where, $\mathbf{x}_{loc}^{i}$ represents the position of the selected point being guided towards exploration, while $\mathbf{x}_{best}^{i}$ signifies the best position attained by this point throughout the optimization process (referred to as the historical best solution). The value of $E^{i}$, which signifies the distance, is employed to establish the extent of the exploration range for this specific point. Consequently, the new solutions are computed using the following equation:

$$\mathbf{x}_{new}^{i}=\mathbf{x}_{loc}^{i}+E^{i}$$

For the remaining portion of the population not chosen for exploration, they are directed to explore the vicinity of the search center, as expressed by the following equation:

$$F^{i}=\mathbf{x}_{loc}^{i}+\mathbf{x}_{best}$$

$$\mathbf{x}_{new}^{i}=\mathbf{x}_{loc}^{i}-rand\times F^{i}$$

In this context, a uniform random value labelled as $rand$ is consistently applied to all design variables. The variable $F^{i}$ represents the distance between the chosen local point and the overall best solution. It's noteworthy that an identical random value is employed for all design variables within each solution. The pseudocode outlining the YUKI algorithm is presented below:

$$\begin{matrix} Load the search parameters \\ Initiate the population\text{ }\mathbf{x} \\ Evaluate the fitness \\ Calculate\text{ }\mathbf{x}_{MeanBest}\text{ }and\text{ }\mathbf{x}_{best} \\ \mathbf{for} \zeta=1 to \zeta_{\max} \\ Calculate the local boundaries \\ Generate the local population randomly \\ \mathbf{if} rand < EXP \\ Calculate the exploration solutions \\ \mathbf{else} \\ Calculate focus solutions \\ \mathbf{end} \\ update\text{ }\mathbf{x}_{MeanBest} \\ update \mathbf{x}_{best} if better solution is found \\ \mathbf{end} \\ return \mathbf{x}_{best} \end{matrix}$$

The MASTO method initiates by adopting the binarized design domain, a process facilitated through the application of the YUKI algorithm ^4^. Following this, the initial design domain undergoes an objective function evaluation, leading to the division of the design space into distinct local and exploration regions. The design variables are partitioned into various versions, each assigned to either the local or exploration spaces. Notably, these discretized elements are devoid of adaptive functions, such as penalization or relaxation terms. This strategic approach enhances the prospects of attaining global extrema, a characteristic that remains significant even when employing single-point search techniques ^1516^.

Different optimization techniques have been utilized to enhance the achievement of optimal solutions and reduce the possibility of ending up in suboptimal states. In the realm of topology optimization, where the problems are often of substantial scale, heuristic methods are particularly important. They serve to decrease computational expenses and bolster the stability of the optimization process. Nonetheless, reaching the most optimal design hinges on various factors, among which are the selection of search increments and the initial point of the search algorithm. The latter aspect holds particular importance, notably in complex functions like the maximization of heat conductivity in point-to-volume approaches ^1174^. Metaheuristic optimization algorithms are well-known for their capacity to address general-purpose optimization problems without necessitating gradient information, simplifying their practical implementation. Furthermore, their distinctive multipoint search strategy distinguishes them from gradient descent methods, facilitating effective exploration of the solution space and the potential identification of global extrema. Notably, the capability of metaheuristics to embrace this multipoint search approach can be advantageous in the context of selecting appropriate initial points for topology optimization. This, in turn, enables the realization of optimal designs with minimized computational expenses ^4^. In this study, our aim is to attain structures that exhibit both elevated heat conductivity and minimal electromagnetic interference (EMI) concurrently. To achieve this dual objective, we adopt an optimization methodology centered on the reduction of the ratio between heat resistance and the Voltage Standing Wave Ratio (VSWR) inherent in the design configuration, as illustrated in Equation (12).

$$\begin{matrix} find \mathbf{x}=[\text{x}_{i=1..N}] \\ \min: f\left(\mathbf{x}\right)=\frac{C}{VSWR} \\ \mathrm{s}.\mathrm{t}.\left\{\int_{\Omega} \mathbf{x} d\Omega\leq V , \mathbf{x} =0|1 \forall\mathbf{x}\in\Omega\right. \\ \end{matrix}$$

To enhance the efficiency of our solution process, we adopt a method that involves exploring multiple initial search points. This optimization strategy begins by generating an initial set of potential solutions, which are initialized based on recommendations from the YUKI algorithm. These solutions are introduced as a collection of initial strip lines within the design domain. The YUKI algorithm evaluates the objective function by exploring various solution spaces, effectively balancing the exploration of diverse regions and the refinement of promising solutions, a process known as exploitation. This dual approach prevents the algorithm from getting trapped in local optima while refining solutions in areas of high potential. This innovative method accelerates the pursuit of the desired structural configuration with the desired attributes. By combining these methodologies, our research aims to create structures with exceptional heat conductivity and minimal EMI, making them versatile for a wide range of applications across various domains.

**References**

1. Yan, S., Wang, F. & Sigmund, O. On the non-optimality of tree structures for heat conduction. *Int. J. Heat Mass Transf.* **122**, 660–680 (2018).

2. Yamada, T., Izui, K., Nishiwaki, S. & Takezawa, A. A topology optimization method based on the level set method incorporating a fictitious interface energy. *Comput. Methods Appl. Mech. Eng.* **199**, 2876–2891 (2010).

3. Takezawa, A., Nishiwaki, S. & Kitamura, M. Shape and topology optimization based on the phase field method and sensitivity analysis. *J. Comput. Phys.* **229**, 2697–2718 (2010).

4. Al Ali, M., Shimoda, M., Benaissa, B. & Kobayashi, M. Non-parametric optimization for lightweight and high heat conductive structures under convection using metaheuristic structure binary-distribution method. *Appl. Therm. Eng.* **233**, 121124 (2023).

5. Allaire, G., Jouve, F. & Toader, A.-M. Structural optimization using sensitivity analysis and a level-set method. *J. Comput. Phys.* **194**, 363–393 (2004).

6. Wadbro, E. & Berggren, M. Megapixel topology optimization on a graphics processing unit. *SIAM Rev.* **51**, 707–721 (2009).

7. Bejan, A. Constructal-theory network of conducting paths for cooling a heat generating volume. *Int. J. Heat Mass Transf.* **40**, 799–816 (1997).

8. Golio, M. *The RF and microwave handbook*. (CRC press, 2000).

9. Al Ali, M. & Shimoda, M. Toward multiphysics multiscale concurrent topology optimization for lightweight structures with high heat conductivity and high stiffness using MATLAB. *Struct. Multidiscip. Optim.* **65**, 1–26 (2022).

10. Al Ali, M. & Shimoda, M. Hygrally activated displacement inverter using a multiphysics multiscale topology optimization with considering evaporation. *Struct. Multidiscip. Optim.* **66**, 1–16 (2023).

11. Lee, G., Lee, I. & Kim, S. J. Topology optimization of a heat sink with an axially uniform cross-section cooled by forced convection. *Int. J. Heat Mass Transf.* **168**, 120732 (2021).

12. Abed, K. A., Khalil, E. E., Abouel-Fotouh, A. M., El-Hariry, G. & Abd El Salam, L. O. Optimal design of a counter flow cooling tower using PSO algorithm for operating cost minimization. *Appl. Therm. Eng.* **143**, 149–159 (2018).

13. Azimifar, A. & Payan, S. Enhancement of heat transfer of confined enclosures with free convection using blocks with PSO algorithm. *Appl. Therm. Eng.* **101**, 79–91 (2016).

14. Amoura, N., Benaissa, B., Al Ali, M. & Khatir, S. Deep Neural Network and YUKI Algorithm for Inner Damage Characterization Based on Elastic Boundary Displacement. in *Proceedings of the International Conference of Steel and Composite for Engineering Structures: ICSCES 2022* 220–233 (2023).

15. Dzierżanowski, G. On the comparison of material interpolation schemes and optimal composite properties in plane shape optimization. *Struct. Multidiscip. Optim.* **46**, 693–710 (2012).

16. Rozvany, G. The SIMP method in topology optimization-theoretical background, advantages and new applications. in *8th Symposium on Multidisciplinary Analysis and Optimization* 4738 (2000). doi:10.2514/6.2000-4738.

17. Iga, A., Nishiwaki, S., Izui, K. & Yoshimura, M. Topology optimization for thermal conductors considering design-dependent effects, including heat conduction and convection. *Int. J. Heat Mass Transf.* **52**, 2721–2732 (2009).
